# Smart Nanoarchitectures for Precision RNA Delivery: Harnessing Endogenous and Exogenous Stimuli in Cancer Treatment

**DOI:** 10.7150/thno.112492

**Published:** 2025-07-02

**Authors:** Jintao Hao, Yue Li, Lu Huang, Nannan Qi, Yanan Sun, Chaoxing He, Shaokun Yang, Zhiyun Niu, Xianrong Qi, Bai Xiang

**Affiliations:** 1 Hebei Key Laboratory of Innovative Drug Research and Evaluation, School of Pharmaceutical Sciences, Hebei Medical University, Shijiazhuang 050017, PR China.; 2 Department of Hematology, The Second Hospital of Hebei Medical University, Shijiazhuang 050000, PR China.; 3 Key Laboratory of Molecular Pharmaceutics and New Drug Delivery System, School of Pharmaceutical Sciences, Peking University, Beijing, 100191, P.R. China.; 4 National Key Laboratory of New Pharmaceutical Preparations and Excipients, Shijiazhuang, 050017, PR China.

**Keywords:** stimuli-responsive, nanocarriers, RNA delivery, cancer treatment, endogenous and exogenous stimuli

## Abstract

RNA therapy holds great potential for cancer treatment owing to its ability to regulate gene expression precisely, thereby inhibiting tumor growth and metastasis. However, RNA delivery faces several physiological challenges, including rapid degradation by nucleases, limited cellular uptake, and inefficient intracellular release. To address these limitations, stimuli-responsive nanocarriers have been developed to enhance RNA delivery and improve therapeutic efficacy while minimizing side effects. These intelligent systems are designed to respond to specific endogenous or exogenous stimuli (*e.g.*, pH, redox potential, enzyme, light, magnetic fields, and ultrasound), enabling targeted delivery and controlled RNA release. This review highlights recent advances in the design and mechanisms of stimuli-responsive RNA nanocarriers, emphasizing key research findings and exploring future perspectives for their clinical translation.

## 1. Introduction

**Figure 1:**
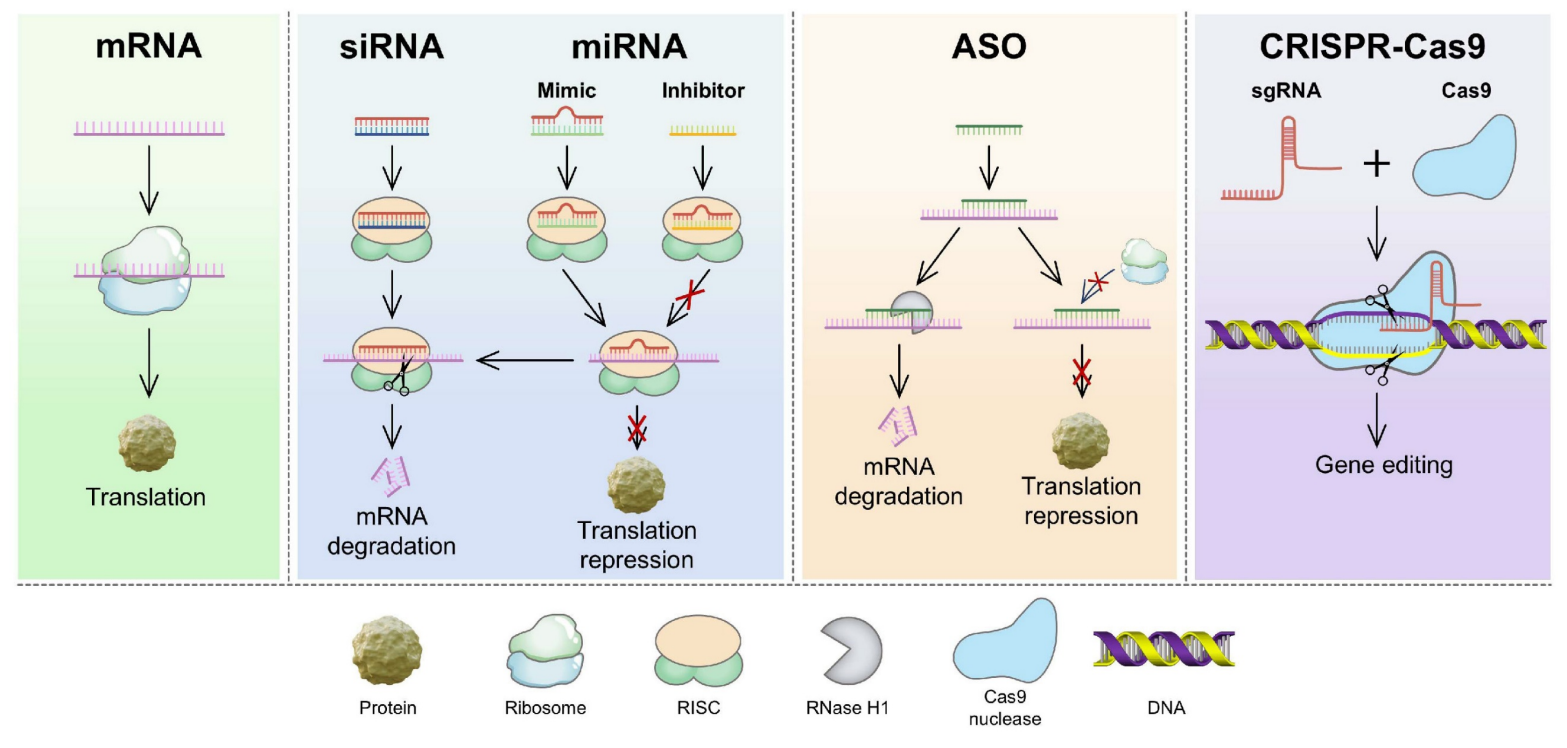
** Mechanisms of RNA therapies from mRNA translation to CRISPR-based genome editing.** mRNA is translated into proteins. siRNA, miRNA mimics, and ASOs mediate mRNA degradation or translation blockade. miRNA inhibitors prevent translation repression. Cas9-sgRNA RNP complexes induce DNA double-strand breaks for precise gene editing. ASO: antisense oligonucleotides; Cas9: CRISPR-associated protein 9; CRISPR: clustered regularly interspaced short palindromic repeats; miRNA: microRNA; mRNA: messenger RNA; RISC: RNA-induced silencing complex; sgRNA: single-guide RNA; siRNA: small interfering RNA.

**Figure 2:**
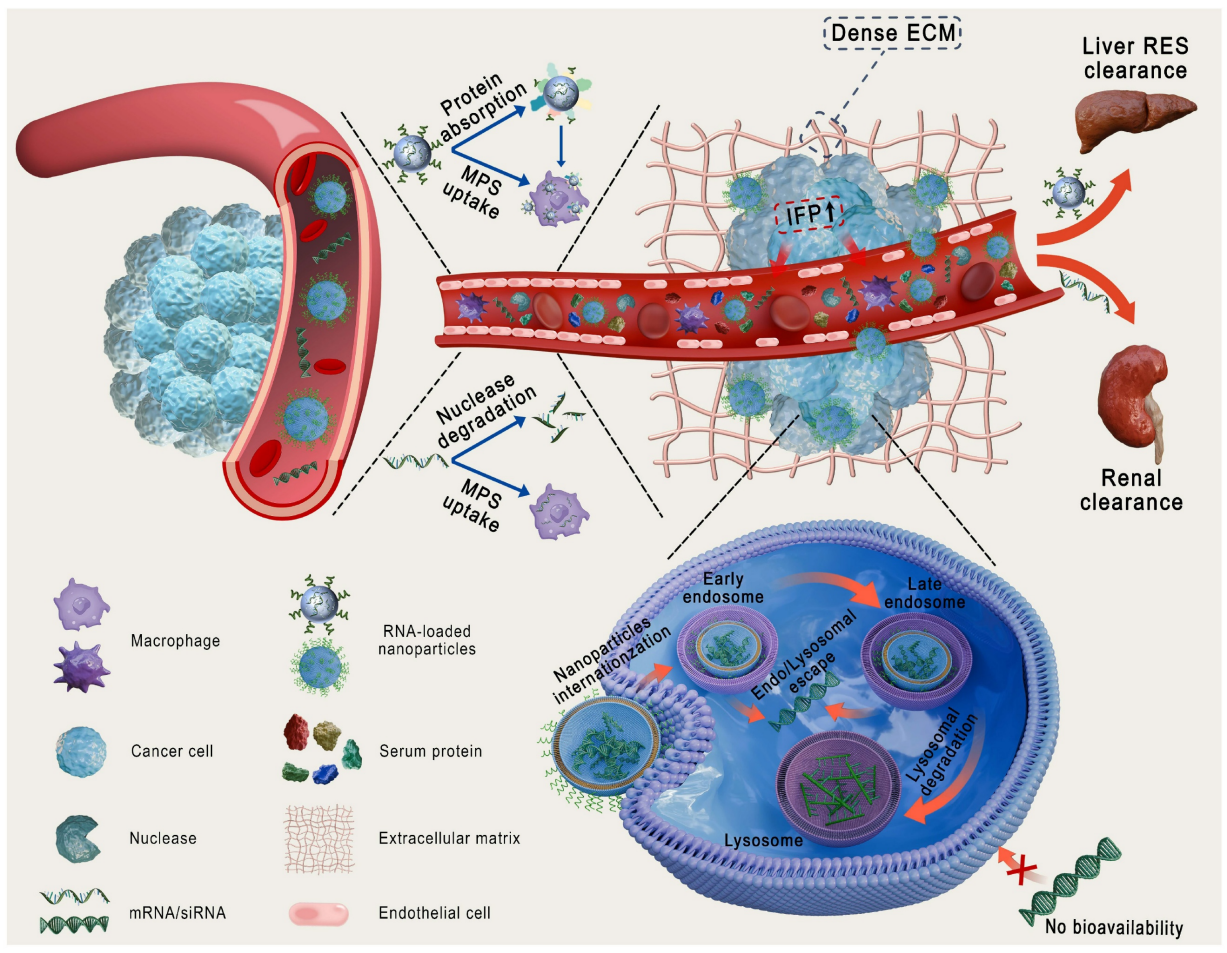
** Physiological barriers of RNA and traditional nanoparticles delivery.** ECM: extracellular matrix; IFP: interstitial fluid pressure; MPS: mononuclear phagocyte system; RES: reticuloendothelial system.

Compared with other therapeutic modalities, RNA-based therapies have several advantages, such as high efficiency, strong target specificity, minimal side effects, and simplified molecular design, making them a focal point in modern biomedical research 1. The primary types of RNA used in cancer treatment include messenger RNA (mRNA), small interfering RNA (siRNA), microRNA (miRNA), antisense oligonucleotides (ASO), and the CRISPR/Cas9 gene-editing system (Figure 1) 2, 3. These RNAs treat diseases or prevent their progression by increasing the expression of specific proteins or suppressing their production 4. However, RNA delivery encounters various physiological challenges (Figure 2), including (1) rapid degradation by circulating nucleases, (2) recognition and clearance by phagocytic cells, (3) rapid renal filtration and elimination, (4) limited membrane permeability due to their high molecular weight and negative charge, (5) inherent immunogenicity that may trigger undesirable immune responses, and (6) lysosomal degradation of internalized RNA 5, 6. Thus, the direct use of free RNA is largely ineffective, necessitating the development of advanced delivery systems to enhance RNA stability, enable tissue-specific targeting, and facilitate efficient intracellular release, thereby maximizing therapeutic efficacy 7.

**Figure 3:**
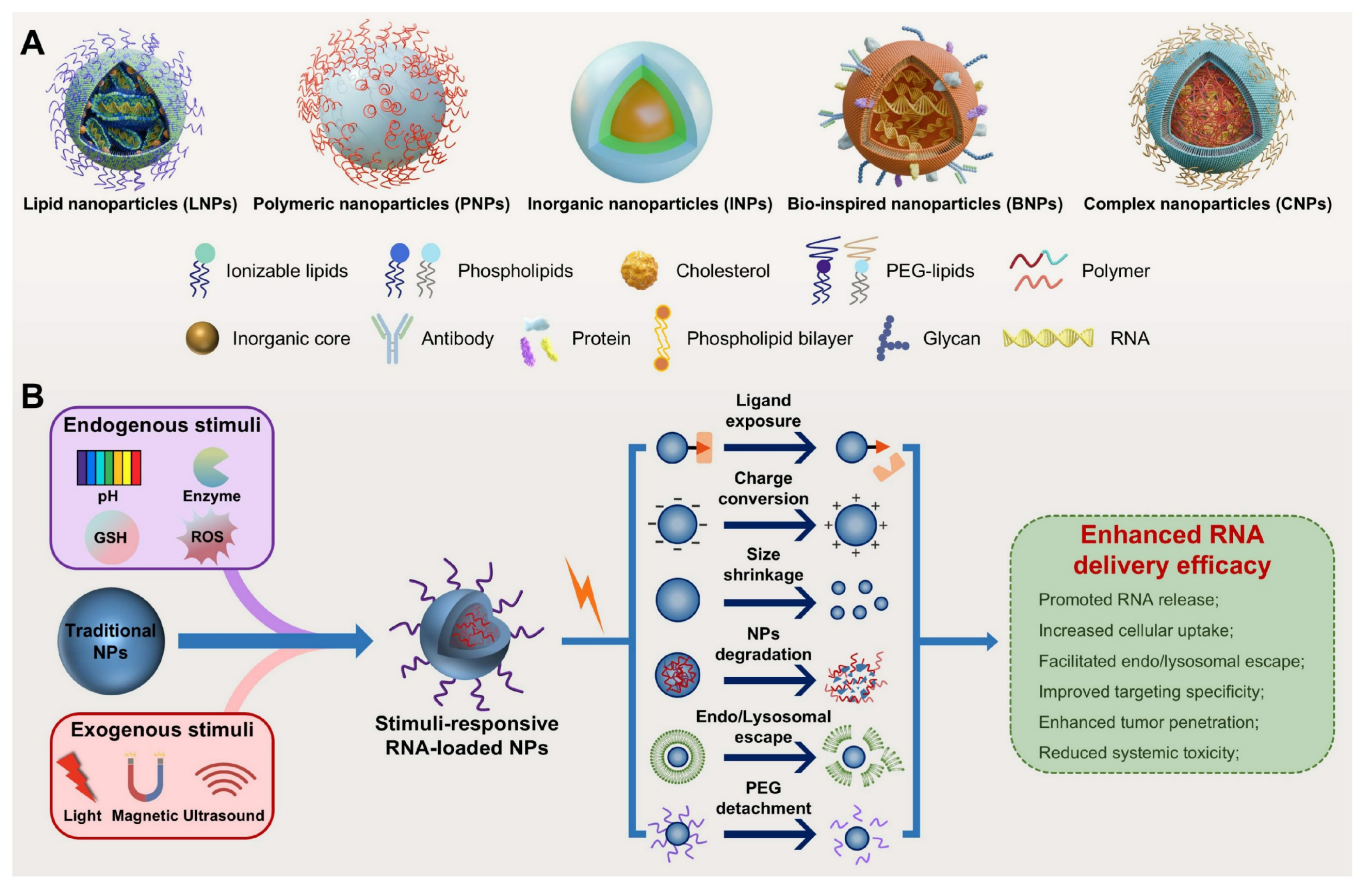
** Schematic illustration of nanoplatforms for RNA delivery.** (A) Representative nanoparticles for RNA delivery. (B) Stimuli-responsive nanoparticles for enhanced RNA delivery: from stimulus-gated design to dynamic response mechanisms. GSH: glutathione; NPs: nanoparticles. PEG: polyethylene glycol; ROS: reactive oxygen species.

Nanoparticles (NPs), including lipid- and polymer-based, inorganic, bio-inspired, and complex NPs (Figure 3A), have been extensively investigated as RNA delivery platforms 1. These systems protect RNA from nuclease degradation, enhance cellular uptake, and facilitate endo-/lysosomal escape, thereby improving RNA delivery 8, 9. However, traditional nanocarriers still face various biological challenges (Figure 2): (1) in circulation, they adsorb serum proteins, forming a "protein corona" that may alter stability and biodistribution; (2) they are easily cleared by the mononuclear phagocyte system (MPS) or reticuloendothelial system (RES); and (3) within the tumor microenvironment (TME), the dense extracellular matrix (ECM) and elevated interstitial fluid pressure (IFP) hinder deep tissue penetration 10, 11. More importantly, these carriers exhibit low targeting efficiency, often resulting in nonspecific release. Furthermore, only a small proportion of internalized carriers successfully escape from endo-/lysosomes, markedly impairing the efficacy of RNA delivery 12. Therefore, traditional nanocarriers often fail to achieve optimal therapeutic outcomes. Intelligent-responsive nanoplatforms are eagerly demanded to overcome these barriers and improve the performance of RNA delivery (Table 1) 13.

Stimuli-responsive nanocarriers, a next-generation delivery platform that enables efficient and targeted nucleic acid delivery by precisely responding to internal or external stimuli, offer a promising strategy to overcome the limitations of traditional nanocarriers (Table 1) 8, 9. Endogenous stimuli include acidic pH, elevated levels of reactive oxygen species (ROS) and glutathione (GSH), overexpressed enzymes, and hypoxia, while exogenous stimuli include light, magnetic fields, and ultrasound (US) 14, 15. These nanocarriers demonstrate high stability under normal physiological conditions but undergo structural or physicochemical changes in response to specific stimuli, markedly enhancing RNA delivery efficacy (Figure 3B) 8, 16. Notably, the ability to precisely control the timing and location of exogenous stimulation enables spatiotemporally regulated RNA release, further improving targeting specificity and reducing toxic side effects 8, 17. In addition, owing to their capacity to sense disease-specific microenvironmental signals, stimuli-responsive platforms have great potential in personalized precision therapy and are expected to facilitate the development of patient-centered, tailored theranostics strategies 18. This review systematically summarizes endogenous and exogenous stimuli-responsive nanocarriers for RNA delivery (Table 2), emphasizing their design strategies, response mechanisms, and biomedical applications. In the field of cancer therapy, this review highlights recent advances and current challenges, offering insights and future directions for the development of safe and effective RNA delivery systems.

## 2. Endogenous Stimuli-Responsive Nanocarriers

**Figure 4:**
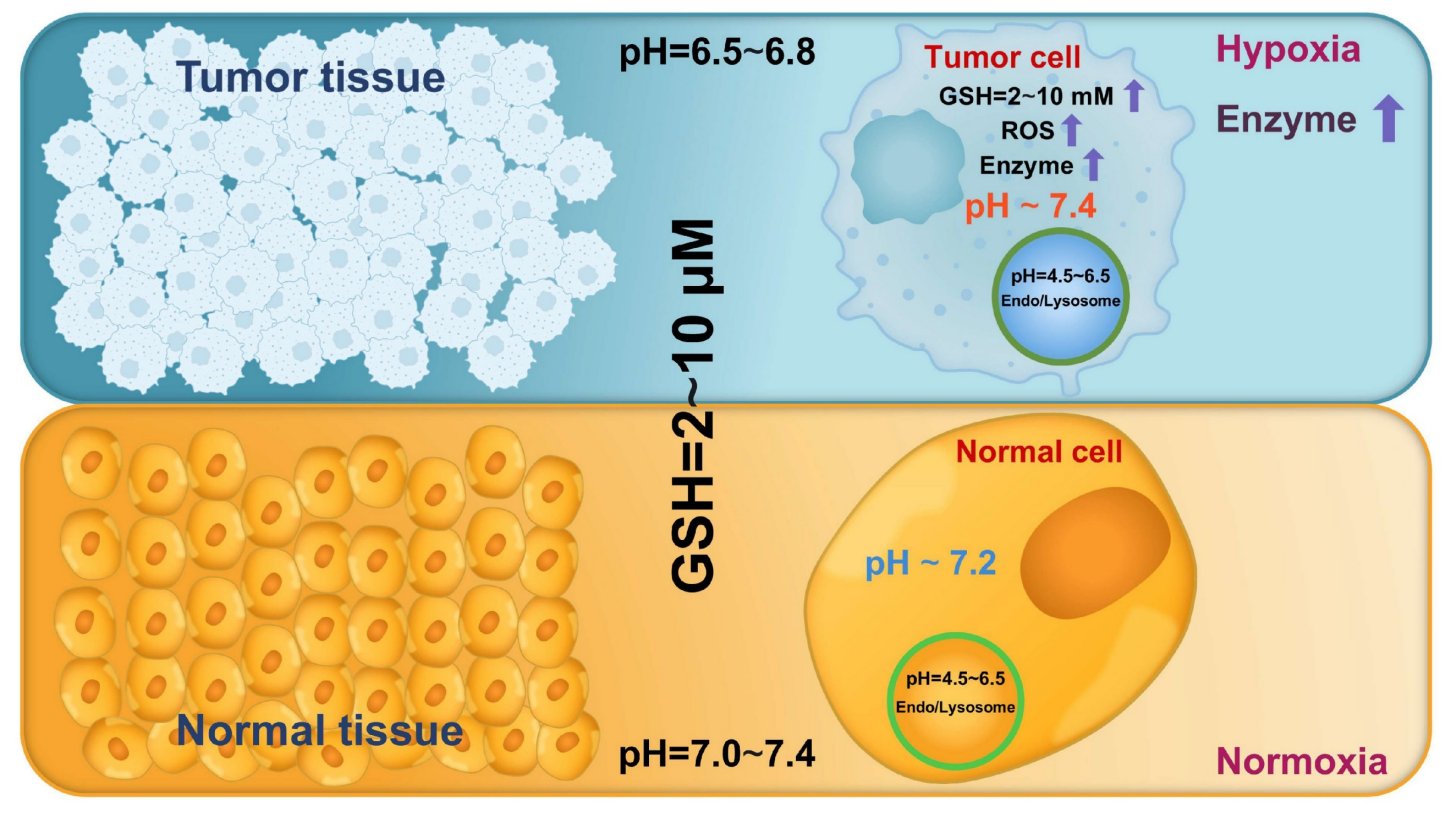
Abnormal biochemical characteristics in the tumor microenvironment and tumor cells.

Cancer has been increasingly recognized as an evolutionary and ecological process that involves continuous dynamic interactions between tumor cells and the TME 49. These interactions collectively promote tumor growth and progression by enhancing cell proliferation, facilitating invasion and metastasis, inducing angiogenesis, and creating an immunosuppressive microenvironment 50. As tumors progress, the biochemical characteristics of the TME and tumor cells markedly differ from those in normal tissues and cells, including lower pH, higher ROS and GSH levels, and overexpressed enzymes (Figure 4) 8. Hence, leveraging these differences to design endogenous stimuli-responsive nanocarriers holds great promise for overcoming biological limitations and achieving precise and efficient RNA delivery 51.

### 2.1 pH-responsive nanocarriers

Altered pH levels are a hallmark of pathological tissues and play a pivotal role in certain processes, such as tumor progression and inflammatory responses 52. Under normal physiological conditions, the pH of human tissues and the blood ranges from 7.0 and 7.4 16. By contrast, pathological regions (*e.g.*, tumors) demonstrate markedly reduced pH values due to enhanced metabolic activity and acidic metabolite accumulation 53. This acidification is largely driven by the "Warburg effect", a metabolic reprogramming phenomenon in which tumor cells mainly rely on glycolysis for energy production even under aerobic conditions 54. This process generates excessive lactic acid, decreasing the pH of the TME to 6.5-6.8 (Figure 4) 14. The intracellular compartments involved in endocytic trafficking also exhibit a pH gradient: the pH of early endosomes ranges from 6.0 to 6.5, progressively decreasing to 4.5-5.0 as they mature into late endosomes and fuse with lysosomes 9. This hierarchical acidification triggers pH-responsive nanocarriers, enabling targeted RNA delivery at the tissue and intracellular levels.

pH-responsive nanocarriers have been developed to enhance the specificity and efficiency of RNA delivery while reducing off-target effects and improving therapeutic outcomes 55, 56. These intelligent delivery systems remain stable under physiological pH, effectively protecting RNA from nuclease degradation 18. However, in acidic environments, they undergo structural or physicochemical changes, which enhance cellular uptake and facilitate endo-/lysosomal escape 8, 57. At present, pH-responsive nanocarriers are mainly designed based on strategies such as acid-labile bond cleavage and protonation/deprotonation of specific chemical groups 14, 58.

#### 2.1.1 Acid-labile bond cleavage

**Figure 5:**
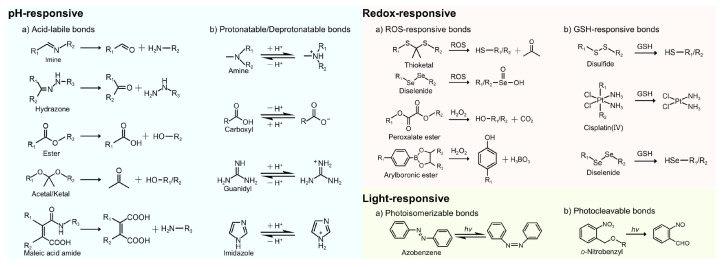
** Stimuli-responsive chemical bonds and their responsive mechanisms.** H_2_O_2_: hydrogen peroxide; *hv*: light irradiation.

Acid-labile chemical bonds, such as hydrazone, imine, ester, acetal/ketal, and maleic acid amide (MAA) derivatives (Figure 5), have been incorporated into nanocarriers. Under acidic conditions, the cleavage of these bonds triggers transformation in nanocarriers, such as charge reversal, size or morphological changes, and structural disruption, thereby enhancing cellular uptake, promoting tumor penetration, and facilitating RNA release 8, 57.

**Figure 6:**
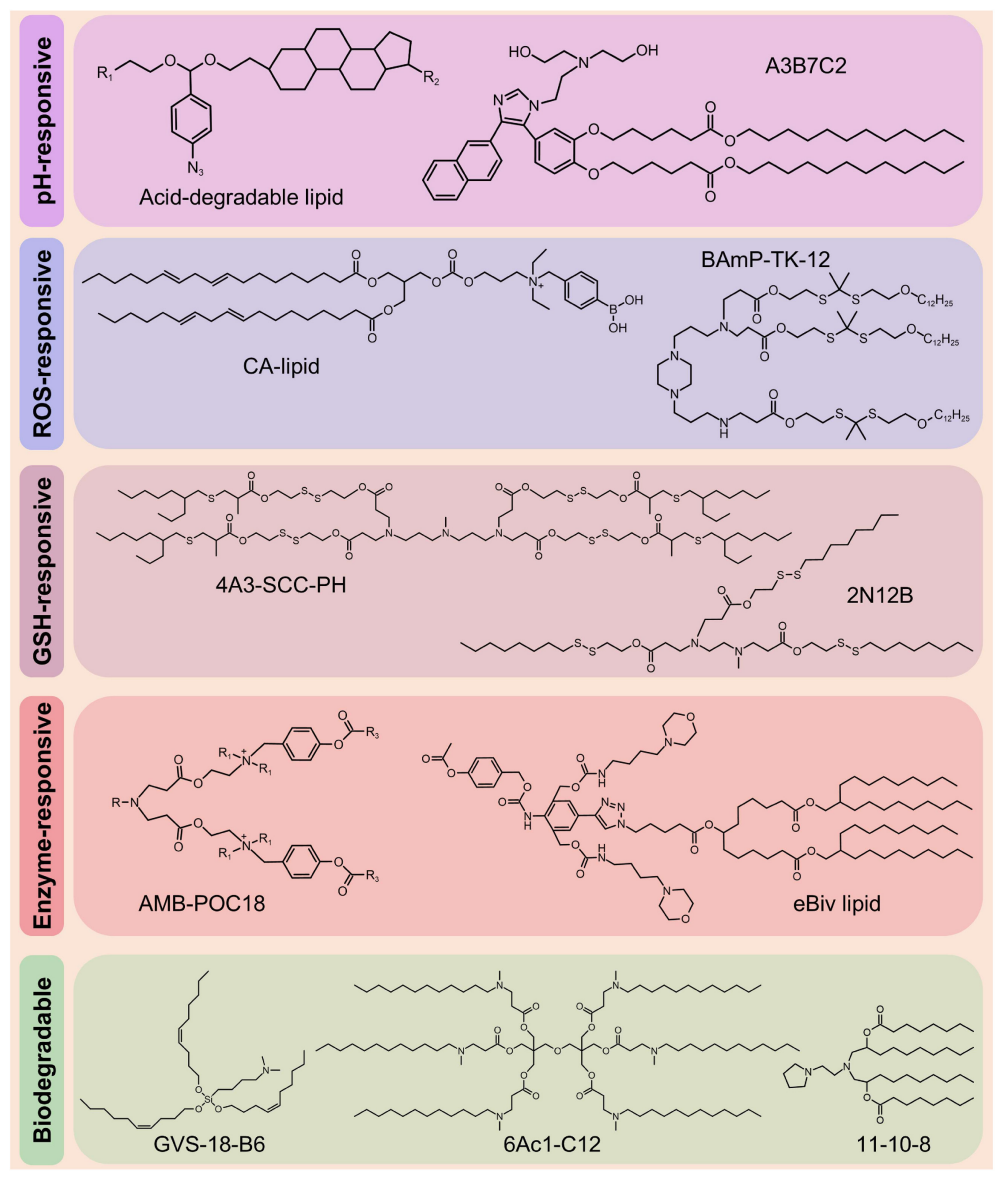
A novel series of engineered ionizable lipids.

Despite the clinical success of LNPs in RNA delivery, their low endo-/lysosomal escape efficiency limits therapeutic efficacy, necessitating higher doses, which, in turn, increases immunogenicity and toxicity risks 59, 60. In addition, the inherent toxicity of cationic and ionizable lipids (ILs), along with their prolonged tissue retention, exacerbates adverse effects 61. These limitations highlight the urgent need for stimuli-responsive nanocarriers that combine efficient RNA delivery with improved biodegradability. Therefore, Zhao *et al.* designed rapidly degradable LNPs (RD-LNPs) incorporating an acid-labile "azido-acetal" linker (Figure 6) enabling pH-responsive hydrolysis (t_1/2_ = 14.8 min at pH 6.0). This rapid hydrolysis enhanced endosomal escape efficiency by 3-fold compared with standard LNPs while reducing NP accumulation and systemic toxicity. The modular platform produced three distinct RD-LNP formulations. ADP-LNPs exhibited multi-organ delivery capabilities, achieving 9-fold and 4-fold higher luc-mRNA delivery to the liver and spleen, respectively, than conventional LNPs and 30% transfection efficiency in the striatum and hippocampus via improved brain diffusion. ADA-LNPs adopted charge-conversion mechanisms, resulting in an 8-fold increase in luciferase activity and ~25% macrophage transfection in the spleen, which are crucial for immunotherapy. ADC-LNPs exhibited lung-specific targeting, with 50% total pulmonary cell transfection and >70% efficiency in the alveolar region, making them suitable for inhalable lung therapeutics 62. Moreover, this platform was used to deliver thermostable iGeoCas9 ribonucleoprotein (RNP) complexes, achieving notable tissue-specific genome editing. ADC-LNPs achieved gene editing in 16% of the total lung tissue and demonstrated 19% editing efficiency of the pathogenic SFTPC gene. These findings indicate that the use of thermostable genome editors is a transformative approach for CRISPR-based therapeutics, enabling targeted delivery that is lacking in conventional nonviral systems and avoiding limitations associated with viral vectors 63. Several groups have recently developed biodegradable ILs, including GVS-18-B6 [64], 11-10-8 [65], and 6Ac1-C12 66 (Figure 6).

The introduction of additional components into LNPs to form hybrid NPs can markedly enhance the efficiency of RNA transfection 59. Zhang *et al.* reported acid-responsive hybrid lipid-polymer NPs (PLNPs) by integrating zwitterionic poly(lactic acid)-block-poly(carboxybetaine) derivatives (PLA-b-PCB-X) into clinically approved Onpattro-LNP formulations. Polymers contain acid-labile hemiacetal ester groups that hydrolyze under acidic endosomal conditions, converting the cationic polymer to a neutral form. This reduces RNA binding affinity and enhances cytosolic release, thereby resulting in a 5.4-fold increase in siRNA silencing efficiency. Notably, although PLNPs exhibited similar cellular uptake and endosomal escape profiles as conventional LNPs, they achieved higher cytoplasmic siRNA concentrations, providing a simple and versatile strategy to enhance the efficiency of RNA delivery 67.

**Figure 7:**
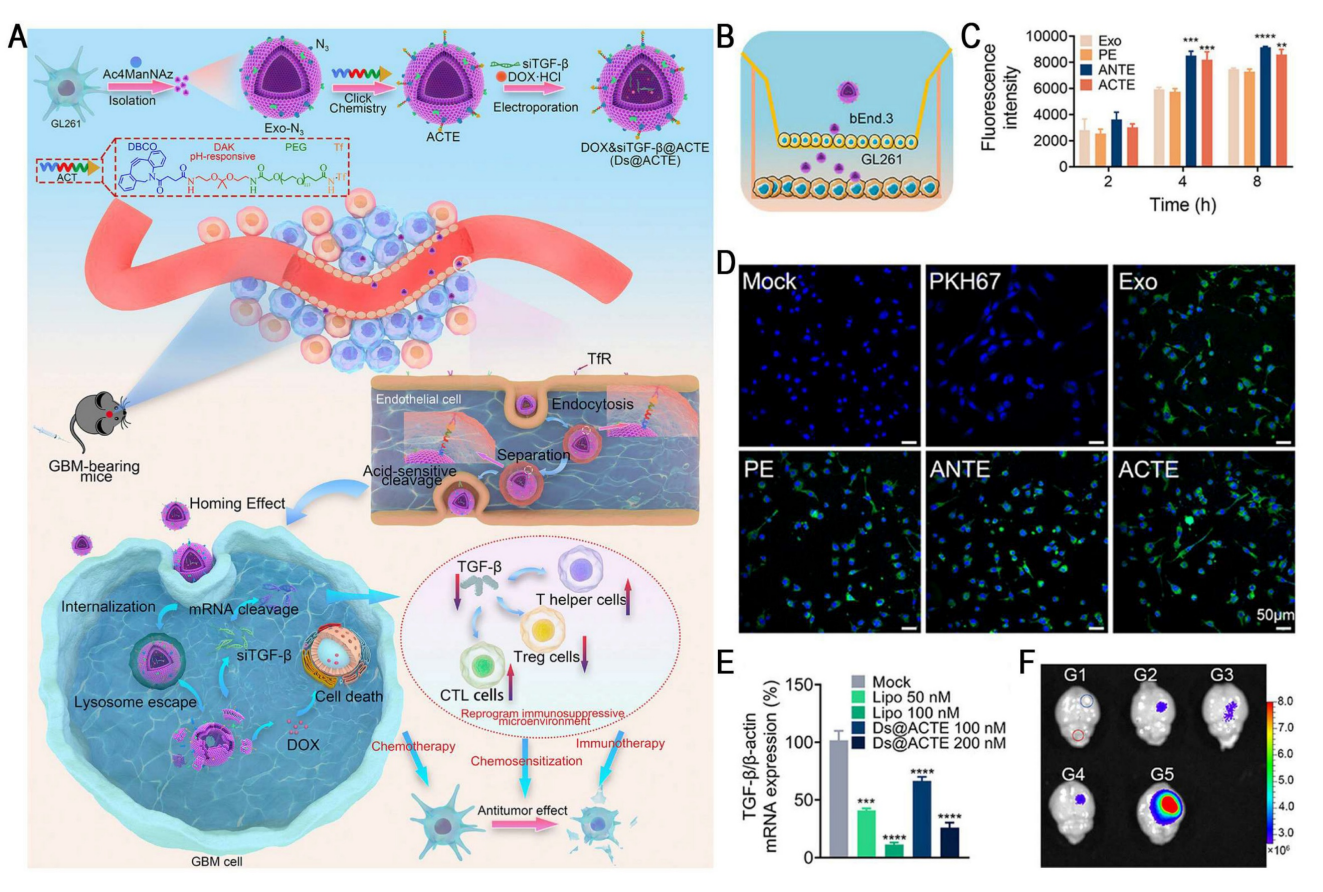
** Acid-cleavable transferrin (Tf)-modified engineered exosome system (Ds@ACTE) to address the challenges of delivering RNA across the BBB.** (A) Schematic illustration of the preparation, GBM-targeting delivery, and chemo-immunotherapy of Ds@ACTE. (B) Schematic illustration of Transwell model. (C) Cellular uptake of different engineered exosomes by bEnd.3 cells after incubation for 2, 4, and 8 h using flow cytometry analysis. (D) Confocal images of GL261 cells in the acceptor chamber of the Transwell model after the introduction of different engineered exosomes for 6 h. Scale bar = 50 µm. (E) Relative expression of TGF-β mRNA in GL261 cells. Cells were treated with Ds@ACTE and siTGF-β@Lipo2000. (F) *Ex vivo* imaging of brains collected at 24 h after injection, blue circle represents glioma site, red circle represents normal brain parenchyma, bar represents radiant efficiency from 2.7 × 10 ^6^ to 8.0 × 10 ^6^ [p s^-1^ cm^-2^ sr^-1^]/[μW cm^-2^]. Adapted with permission from 69, an open access article under a CC-BY 4.0 license. Copyright 2024, The Authors.

Studies have demonstrated a critical tradeoff in transferrin receptor (TfR)-mediated blood-brain barrier (BBB) penetration strategies: high-affinity ligands, such as transferrin (Tf), tend to remain trapped within the endo-/lysosomal compartments of brain endothelial cells due to their strong bond with TfR, which markedly restricts parenchymal delivery. Conversely, low-affinity antibodies improve the efficiency of BBB transcytosis by more rapidly detaching from TfR, leading to markedly increased brain accumulation 68. Therefore, designing intelligent nanocarriers capable of "high-affinity capture at the vascular side and controlled intracellular dissociation" has emerged as a key strategy for overcoming the major bottlenecks in BBB delivery. Yang *et al.* reported an acid-cleavable, Tf-modified engineered exosome system (Ds@ACTE) that incorporates a diamino ketal (DAK) linker to co-deliver siTGF-β and doxorubicin (Dox) (Figure 7). This ligand modification enhanced the efficiency of endothelial cellular uptake, followed by acid-triggered cleavage of the DAK linker within the endo-/lysosome, facilitating efficient Tf-TfR complex dissociation. The separated exosomes exhibited enhanced endo-/lysosomal escape and transcytosis, ultimately achieving superior BBB penetration. Furthermore, GL261-derived exosomes (Ds@Exo) demonstrated inherent homing capability toward glioblastoma (GBM) cells, resulting in a 1.64-fold glioma-to-normal brain accumulation. This dual-functional platform effectively reprogrammed the immunosuppressive TME, enhanced anti-GBM immunity, and synergistically improved the efficacy of chemotherapy, representing a promising combinatorial strategy for GBM therapy 69.

NP PEGylation, a fundamental surface engineering strategy in nanomedicine, leverages hydrophilic PEG chains to reduce plasma protein adsorption and avoid recognition by the MPS, thereby enhancing NP stability and prolonging the systemic circulation time 70. However, this "stealth" property leads to the so-called "PEG dilemma", where PEGylation simultaneously impedes cellular uptake and endo-/lysosomal escape, thereby limiting RNA delivery efficiency 71. Emerging solutions use acid-labile PEG derivatives that selectively detachment in the TME, enhancing NP-cell interactions while preserving systemic "stealth" properties 20. Dong *et al.* designed a TME-responsive nanoplatform (D_m_-NPs) to reverse trastuzumab resistance in HER2-positive breast cancer. The system used acid-labile copolymers (Meo-PEG-Dlink_m_-PLGA) containing 2,3-dimethylmaleic acid (DMA) linkers co-formulated with the cationic lipid G0-C14 for an efficient PTEN mRNA encapsulation (80%). Upon reaching the acidic TME, DMA cleavage induced PEG detachment, which enhanced cellular uptake by 4-fold compared with physiological pH and facilitated endo-/lysosomal escape. This platform exhibited robust transfection efficacy, achieving >80% EGFP expression in trastuzumab-resistant cells and restoring PTEN protein expression by 5-fold. PTEN upregulation effectively suppressed PI3K/Akt pathway hyperactivation, reducing the half-maximal inhibitory concentration (IC_50_) of trastuzumab by 5- or 16-fold in resistant models. The combination of PTEN Dm-NPs and trastuzumab limited tumor growth to less than 3-fold and markedly outperformed either treatment alone. In future, coordinated efforts to optimize manufacturing processes are essential to enhance the stability and RNA-loading consistency of TME-responsive polymeric NPs 72.

#### 2.1.2 Protonation/deprotonation of chemical groups

Another promising strategy for designing acid-responsive nanocarriers is the introduction of ionizable chemical groups (*e.g.*, amine and carboxyl groups) (Figure 5) 73. These groups undergo pH-dependent protonation/deprotonation, which can trigger changes in physicochemical properties or structures 57, 74. For example, the protonation of amine groups induces charge reversal, which improves electrostatic interactions with negatively charged cell membranes. Moreover, it facilitates endo-/lysosomal escape via the "proton sponge effect", thereby improving transfection efficiency 58, 75. Conversely, carboxyl group deprotonation can trigger hydrophilic-to-hydrophobic phase transitions, facilitating RNA release through NP disassembly 74.

pH-responsive polymeric systems have been widely used for RNA delivery 76-78. Polyglutamic acid-polyethylene glycol (PGA-PEG) has been extensively investigated for RNA delivery owing to its dynamic charge-switching behavior. At the physiological pH, the carboxyl groups of PGA are deprotonated to COO^-^, exhibiting high hydrophilicity and a random coil conformation. Under an acidic TME, PGA is protonated to COOH and transforms into hydrophobic α-helix conformation, thereby facilitating RNA release 79. Accordingly, Li *et al.* developed an innovative pH-responsive nanoplatform (CB+miR+R/PGA-SLN-CSW) coated with PGA-PEG layers for the co-delivery of miR-142, immunogenic cell death (ICD) inducers (CB-5083), and an immunoadjuvant (resiquimod), with the aim of achieving an effective treatment for pancreatic ductal adenocarcinoma (PDAC). In the TME, PGA protonation triggered detachment of the outer PGA-PEG shell, exposing surface-conjugated peptides and reversing the NP charge from -4.34 to +15.16 mV, which markedly enhanced uptake by pancreatic cancer cells. This multifunctional NP suppressed cancer cell progression in Panc-02 cells by inhibiting epithelial-mesenchymal transition (EMT), promoting autophagy and apoptosis, and repolarizing M_2_ to M_1_ macrophages 80.

**Figure 8:**
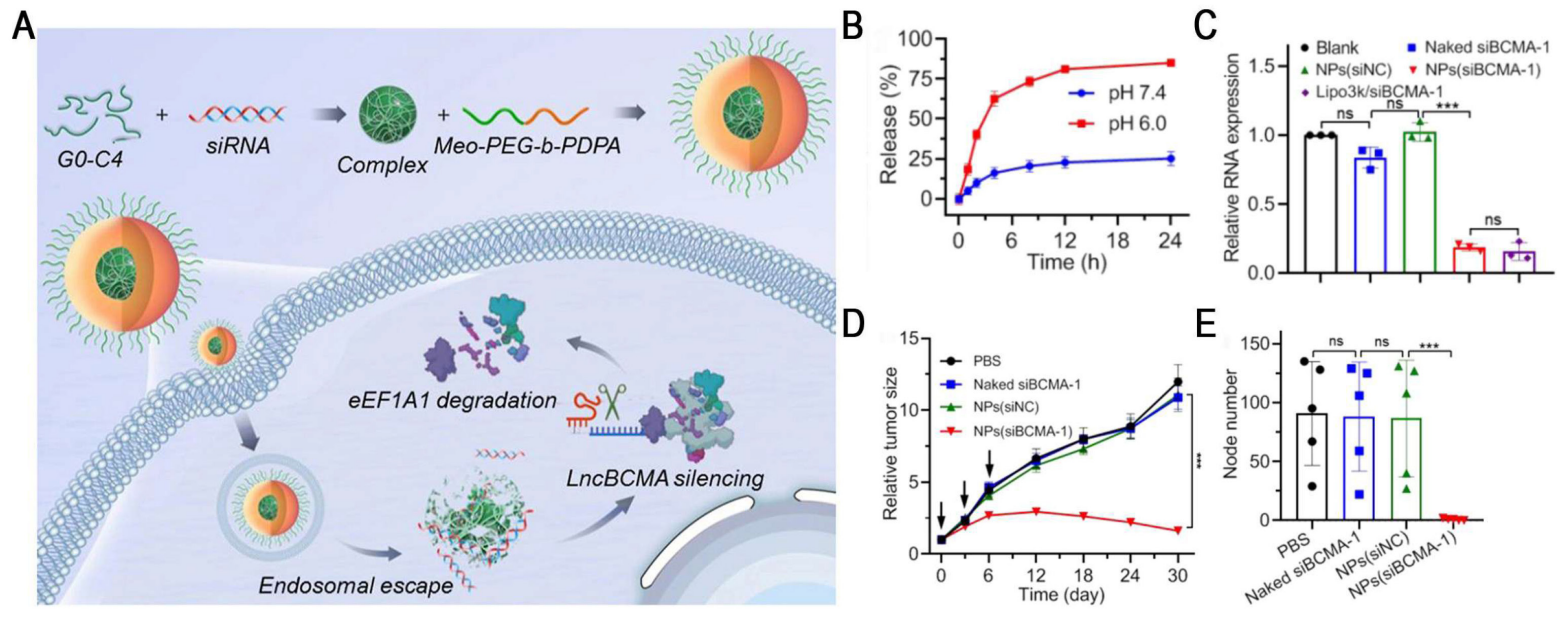
**NPs-mediated lncBCMA silencing to promote eEF1A1 ubiquitination and suppress tumor growth and metastasis.** (A) Schematic illustration of the NPs(siBCMA-1) made with the endosomal pH-responsive polymer Meo-PEG-b-PDPA and cationic lipid G0-C14. (B) The profile of siBCMA release from the NPs(siBCMA-1) incubated in PBS solution at different pHs. (C) qRT-PCR analysis of lncBCMA expression. (D) Tumor growth of MDA-MB-231 orthotopic tumor-bearing mice treated with PBS, naked siBCMA-1, NPs(siNC), and NPs(siBCMA-1). The intravenous injections are indicated by black arrows. (E) Number of metastatic nodes counted from H&E staining of the lungs of MDA-MB-231 orthotopic tumor-bearing mice after treatment with the formulas shown in (D). Adapted with permission from 83, an open access article under a CC-BY 4.0 license. Copyright 2023, Chinese Pharmaceutical Association and Institute of Materia Medica, Chinese Academy of Medical Sciences.

Ultra-pH-sensitive (UPS) nanosystems exhibit a unique binary on/off response to pH, enabling rapid transitions between monomers and micelles within a narrow pH range (ΔpH < 0.5) 81. For example, the representative polymer Meo-PEG-PDPA_80_ has a pKa of ~6.3, which is close to the endosomal pH. In the acidic endosomal environment, PDPA segments undergo protonation, inducing NP disassembly and promoting endo-/lysosomal escape, thereby markedly enhancing the efficiency of RNA delivery 82. Based on this, Yang *et al.* developed an endosomal UPS nanoplatform by co-assembling Meo-PEG-b-PDPA with siBCMA/G0-C14 complexes for the targeted therapy of triple-negative breast cancer (TNBC) (Figure 8). Once in the acidic endosome, the system facilitated efficient siRNA release (>80%) and resulted in ~80% downregulation of lncBCMA expression in MDA-MB-231 cells. lncBCMA silencing promoted the ubiquitination of eEF1A1, thereby markedly suppressing TNBC growth and metastasis 83. Furthermore, PC7A, a polymer in the UPS library, has been demonstrated to activate the stimulator of interferon genes (STING), thereby exerting potent tumor immunotherapeutic effects 84. Based on this property, a multivalent STING nanoagonist (ONM-501) was developed, which uses PC7A micelles to deliver cGAMP, thereby inducing "burst" and "sustained" dual STING activation. This system has shown potent antitumor efficacy in multiple murine tumor models and high tolerability in rodents and non-human primates. ONM-501 is currently undergoing a phase I clinical trial (NCT06022029) in patients with advanced solid tumors and lymphomas to evaluate its safety and therapeutic potential. In future, the integration of PC7A-based platforms with RNA therapeutics and their rational design, endowing them with the capabilities of STING activation and site-specific mRNA delivery, holds great potential for advancing cancer immunotherapy.

LNPs have emerged as one of the most advanced non-viral delivery platforms for RNA therapeutics, with several clinically approved applications 85. Onpattro is the first siRNA-LNP treatment for hATTR amyloidosis, paving the way for the clinical development of LNP-based nucleic acid delivery platforms. Meanwhile, the mRNA-LNP vaccines Comirnaty and Spikevax demonstrated an efficacy of >90% in phase III trials, representing a major achievement in the field of vaccinology and RNA delivery and completely changing the way of responding to the epidemic. These milestones have established a solid foundation for the broader clinical application of LNP-based RNA therapeutics. In a phase IIb trial for resected melanoma, mRNA-4157/V940 (NCT03897881), a personalized cancer vaccine developed by Moderna and Merck, achieved a 49% reduction in recurrence or death and a 62% reduction in distant metastasis or death when combined with pembrolizumab and has thus progressed to phase III trials. NTLA-2001 (NCT04601051), the first *in vivo* CRISPR therapy that uses LNPs to deliver Cas9 mRNA and sgRNA, achieved ~87% reduction in durable serum TTR at a single dose, marking a breakthrough in genome editing. In addition, MT‑302 (NCT05969041), the first intravenously administered *in vivo* mRNA‑CAR drug, was developed to treat TROP2-positive solid tumors and is currently undergoing a phase I clinical trial. Collectively, these examples highlight the growing translational promise of LNP-based RNA technologies across vaccines, gene editing, and adoptive immunotherapy.

The core functional component of LNPs is IL, which exhibits a pH-sensitive behavior. At the physiological pH, ILs remain neutral, thereby markedly reducing nonspecific interactions and systemic toxicity 86. In acidic endo-/lysosomes, these lipids become protonated and acquire positive charges, which promote endo-/lysosomal escape by inducing membrane destabilization and "proton sponge effect", thereby facilitating efficient cytoplasmic RNA delivery 87, 88. Notably, this pH responsiveness has been closely associated with enhanced transfection efficiency and improved therapeutic efficacy in various preclinical models. Recent advancements have focused on the rational design of next-generation ILs guided by structure-activity relationship (SAR) studies, aiming to achieve high safety and tissue-specific delivery (particularly extrahepatic targeting). Naidu *et al.* designed and synthesized an IL library using various biodegradable linkers (*e.g.*, ester, carbonate, amide, and urea) to explore the role of linker chemistry in modulating the organ-targeting specificity of LNPs. Notably, lipid 35 (pKa = 7.67) and lipid 34 (pKa = 7.14), which contain urea or amide linkers, significantly enhanced LNP accumulation in the lungs. SAR analysis revealed that the incorporation of nitrogen atoms into the lipid linker enables fine-tuning of the pKa of LNPs, thereby achieving high protein expression levels selectively in the lungs. They systematically elucidated how linker structural changes mediate organ-selective targeting via pKa regulation, providing essential molecular design principles and theoretical foundations for the development of extrahepatic RNA delivery systems 89.

Selective organ targeting (SORT) technology enables precise organ-specific RNA delivery by adding supplemental components into conventional LNPs. For example, DOTAP, 18PA, and AA11 have been demonstrated to redirect RNA delivery to the lungs, spleen, and bone marrow in mice, respectively 90, 91. Recently, Tang *et al.* synthesized a modular library of lysine-histidine-based lipopeptides (KH-LPs) and constructed a novel lipopeptide-based organ-specific targeting (POST) LNP strategy. The resulting POST-LNPs exhibited higher specificity and efficiency in targeted organs (*e.g.*, lung, liver, and spleen) than the corresponding SORT LNPs. Moreover, SAR analysis revealed that different lipid systems exhibit distinct preferences for KH-LP selection to achieve optimal synergistic effects for an effective and selective RNA delivery. Overall, this strategy provides a universal delivery platform for gene therapy and opens new avenues for extrahepatic RNA delivery 92.

Structural optimization of lipids can significantly enhance RNA delivery. Padilla *et al.* developed a class of branched endosomal disruptor (BEND) lipids using a modular synthetic approach, which considerably improved the efficiency of mRNA and RNP delivery. Experimental results confirmed that terminal branched ILs enhanced endosomal escape, with BEND ILs inducing 2-to-3-fold greater artificial endosome disruption. Notably, these ILs also improved hepatic Cas9 RNP complex delivery and enhanced T cell transfection, showing the versatility of these lipids. In addition, the study showed that even a single methyl group variation in the lipid structure can markedly influence delivery performance, providing an important example for lipid SAR analysis 93. With the advancement of technology, researchers have developed a series of novel ILs, including A3B7C2 (Figure 6) 94, H1L1A1B3 95, and L17-F05 96.

However, the development of existing ILs is hindered by complex chemical design and inefficient screening strategies, substantially impeding the clinical translation of RNA-LNPs. Machine learning (ML) has recently attracted widespread attention as a tool for assisting the design of LNP. Li *et al.* integrated ML with combinatorial chemistry to accelerate IL development. First, they constructed a library of 584 ILs using a high-throughput synthesis (HTS) platform based on the Ugi four-component reaction (4-CR) and trained ML models with structural data and mRNA transfection results. Second, they selected the best-performing XGBoost model to screen a virtual library of 40,000 lipids. Finally, they identified a structurally unique lipid, 119-23, which outperformed the current commercial benchmark lipid, namely, MC3, in muscle and immune cell transfection, demonstrating great potential for immunotherapy 97. Similarly, Witten *et al.* reported deep learning (DL)-based lipid optimization using neural networks (LiON) system, achieving a breakthrough in pulmonary gene delivery. They collected 9,302 datapoints from LNP activity measurements and used them to train a directed message-passing neural network to predict the efficiency of nucleic acid delivery with diverse lipid structures. Using the LiON platform, they evaluated 1.6 million candidate lipids from the 4-CR library and identified two novel structures, the FO-32 and FO-35, which achieved exceptional mRNA delivery to the mouse muscle, lung, and nose. When administered via nebulization, the FO-32 and FO-35 LNPs achieved efficient transfection in ferret lungs, highlighting their potential for pulmonary gene therapy. Overall, they used a "structure extrapolation-activity prediction" strategy to advance the application of artificial intelligence (AI) and DL in improving NP delivery, making a substantial contribution to the development of intelligent gene delivery systems 98. In summary, AI, ML, and DL play pivotal roles in the development of smart nanodelivery systems 99, 100. The application of these technologies enhances the accuracy and efficiency of IL design and is expected to accelerate the clinical translation of stimuli-responsive nanocarriers.

Although pH-responsive nanocarriers hold considerable potential for enhancing RNA delivery efficiency, the TME exhibits substantial heterogeneity in acidity, with some regions demonstrating severe acidification (pH < 5.3). This variability can cause premature carrier degradation or RNA pre-release, thereby reducing targeted delivery efficiency and exacerbating uneven RNA distribution across tumor regions. Furthermore, complex manufacturing processes and the risk of off-target activation *in vivo* have further hindered its clinical translation. To address these challenges, future research should prioritize (1) developing smart delivery platforms that integrate multiple stimulus-responsive elements and synergistic therapeutic modules to improve RNA delivery; (2) establishing standardized, organoid-based evaluation systems to simulate response thresholds, release kinetics, and nanocarrier safety, supporting the creation of accurate predictive models; and (3) using microfluidic technologies to enhance the controllability and batch-to-batch consistency of NP fabrication, allowing for GMP-compliant large-scale production. Notably, the deep integration of material chemistry and synthetic biology promotes the development of novel smart carriers (*e.g.*, DNA origami, metal-organic frameworks and spherical nucleic acids), which are expected to shift RNA delivery systems from a "passive response" to an "active adaptation", ultimately advancing personalized cancer treatment in the context of precision medicine.

### 2.2 Redox-responsive nanocarriers

Tumor tissues exhibit distinct redox dysregulation characterized by higher ROS and GSH levels compared with normal tissues 101. Increased ROS levels result from the aberrant metabolism and mitochondrial dysfunction of tumor cells. Although excessive ROS can suppress proliferation and induce apoptosis under extreme oxidative stress, tumor cells activate intracellular antioxidant systems to maintain redox homeostasis and support survival 102, 103. GSH plays a crucial role in this regulation, with elevated levels consistently observed in various tumor cells 104. This pathological imbalance highlights the need to develop redox-responsive nanocarriers that incorporate redox-sensitive chemical bonds 105. These systems undergo specific structural or chemical transformations, thereby promoting nanocarrier degradation and RNA release 106, 107. This strategy holds great potential in enhancing the efficacy and specificity of cancer treatment.

#### 2.2.1 ROS-responsive nanocarriers

ROS mainly include superoxide (O_2_^•-^), hydroxyl radical (•OH), hydrogen peroxide (H_2_O_2_), and singlet oxygen (^1^O_2_) 101. Among these, H_2_O_2_ is the most abundant ROS in eukaryotes owing to its relatively long half-life 108. The concentration of H_2_O_2_ is markedly elevated in tumor tissues (50-100 μM), considerably exceeding those observed in normal tissues (~20 nM) 18. The current strategies for ROS-responsive nanocarriers predominantly exploit four types of chemical bonds, namely, peroxalate esters, diselenides, thioketals, and arylboronic acids/esters (Figure 5) 109.

Peroxalate esters and arylboronic acids/esters demonstrate exceptional sensitivity to H_2_O_2_. Consequently, Yang *et al.* developed an H_2_O_2_-responsive charge-altering LNP (CALNP) platform for an efficient siRNA delivery (Figure 6). By modifying ILs with phenylboronic acid (PBA), they permanently synthesized cationic lipids (CA-lipid 5) that exhibit dual-targeting mechanisms. First, PBA selectively binds to sialic acid (SA) overexpressed on tumor cell membranes, enhancing cellular uptake by ~1.7- and ~2.4-fold compared with non-charge-altering LNPs (nCALNPs) and ionizable LNPs (iLNPs). More importantly, elevated intracellular H_2_O_2_ levels induce PBA cleavage into phenol derivatives, converting CALNPs into iLNPs with reduced positive charge. This transformation facilitated cytoplasmic siRNA release, resulting in a gene-silencing efficiency of ~96%, outperforming Lipofectamine (Lipo) 2000. Furthermore, CALNPs exhibited prolonged tumor retention (6-day fluorescence persistence) and markedly suppressed tumor growth. Owing to their ability to balance circulatory stability and achieve efficient siRNA delivery, CALNPs represent a promising platform for RNA-based cancer therapeutics 110.

Jing *et al.* reported pH/ROS dual-responsive nanocomplexes (MiR@PCPmPs NPs) to reverse the immunosuppressive TME in TNBC. The nanoplatform was created using mannose-functionalized copolymers (PEG-CDM-PEI[Man]-ox-PCL) containing ROS-cleavable peroxalate ester (ox) bonds, achieving a miR155 encapsulation efficiency of >95%. After extravasation into the acidic TME, carboxydimethyl maleate (CDM) cleavage induced PEG detachment and mannose exposure, thereby enhancing uptake by tumor-infiltrating dendritic cells (TIDCs) and tumor-associated macrophages (TAMs). Intracellular ROS further promoted nanocomplex disassembly via ox cleavage, resulting in >70% miR155 release within 4 h. This dual-responsive system reprogrammed the immune microenvironment by activating TIDCs and repolarizing TAMs toward M_1_-like phenotypes. Notably, it increased CD8^+^ T cell infiltration while reducing immunosuppressive cells, including myeloid-derived suppressor cells (MDSCs) and regulatory T cells (Tregs), ultimately establishing durable antitumor immune memory. This platform markedly inhibited primary tumor growth and reduced lung metastasis, providing a transformative approach to boosting antitumor immunity in TNBC and similar malignancies 111.

**Figure 9:**
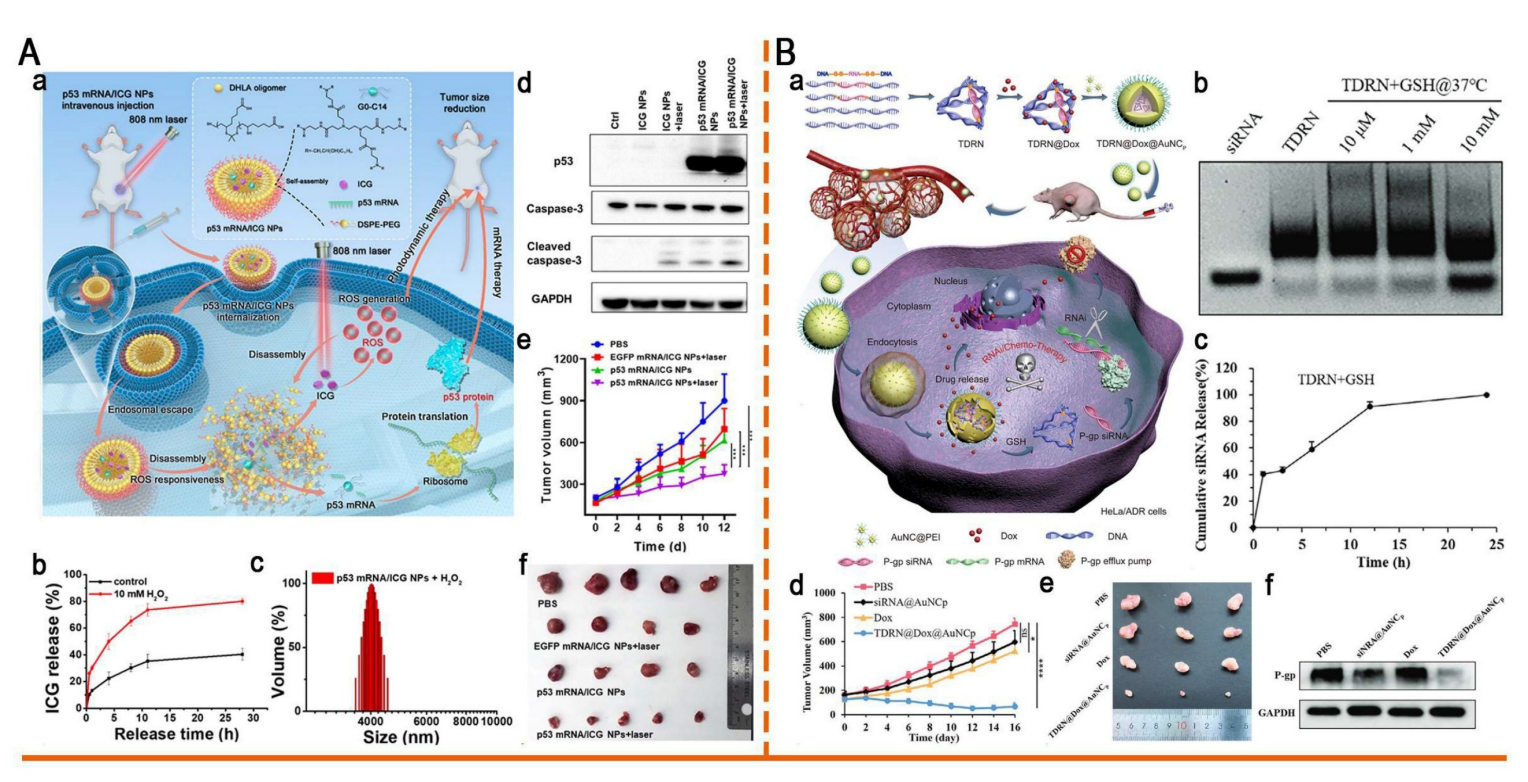
** Redox-responsive nano-delivery systems for RNA therapy.** (A) ROS-responsive nanoparticle delivery of mRNA and photosensitizer for combinatorial cancer therapy. (a)Schematic illustration of ROS-responsive p53 mRNA/ICG NPs for combinatorial cancer therapy. (b) Release kinetics of ICG from p53 mRNA/ICG NPs in the presence vs absence of H_2_O_2_. (c) DLS of p53 mRNA NPs after 2 min of incubation in 10 mM H_2_O_2_. (d) Western blot analysis of p53, caspase-3, and cleaved caspase-3 from different groups *in vitro*. GAPDH was used as the loading control. (e) Tumor growth profile of each indicated treatment group. (f) Photograph of tumor tissues obtained in different groups at day 12. Adapted with permission from 112, copyright 2023, American Chemical Society. (B) Programmable tetrahedral DNA-RNA nanocages woven with GSH-responsive siRNA for enhancing therapeutic efficacy of multidrug-resistant tumors. (a) Schematic illustration of DNA-RNA nanocages for synergistic RNAi/Chemo-therapy of multidrug-resistant tumors. (b) The TDRN was incubated with different GSH concentrations at 37°C for 24 h and analyzed on 1.5% agarose gels. (c) Percentages of cumulative siRNA release from the TDRN with 10 mM GSH solution were calculated using Image J software. (d) Tumor volumes of HeLa/ADR tumor-bearing mice during 16 days of observation. (e) Photograph of excised tumor tissues at day 16. (f) Western blot analysis of P-gp protein expression in excised tumor tissues after treatment with PBS, siRNA@AuNCp, free Dox, and TDRN@Dox@AuNCp. Adapted with permission from 121, an open access article under a CC-BY 4.0 license. Copyright 2024, The Authors.

In addition, thioketal and diselenide bonds have been incorporated into the design of ROS-responsive nanomaterials. For the first time, Zhou *et al.* developed a ROS-responsive polymeric nanoplatform (p53 mRNA/ICG NPs) for the co-delivery of mRNA and photosensitizers (PS) (Figure 9A). This system was self-assembled from thioketal-containing o-DHLA oligomers and lipids. Exposure to ROS induced thioketal bond cleavage, which led to the disassembly of NP and the release of p53 mRNA and indocyanine green (ICG). Upon 808 nm laser irradiation, ICG generated additional ROS, which not only induced photodynamic therapy (PDT) but also accelerated NP disassembly, thereby enhancing p53 mRNA translation and synergistically inhibiting lung cancer progression 112. Gao *et al.* reported a ROS-responsive diselenide-crosslinked polypropyleneimine (PEI) nanogel (A_1.8_Se_3_O_0.5_/siPD-L1). This nanogel facilitated efficient siRNA release via ROS-induced cleavage of Se-Se bonds and leveraged PEI-mediated endosomal escape. By silencing PD-L1 and upregulating MHC-I expression, this system significantly enhanced antitumor immunity and effectively suppressed the growth of colorectal cancer, providing a new strategy for combining autophagy modulation with immune checkpoint blockade 113.

Tellurium demonstrates higher sensitivity to ROS than that of sulfur and selenium owing to its lower electronegativity, making it an ideal candidate for ROS-responsive drug delivery systems 114. Dang *et al.* developed a hierarchical ROS-responsive core-shell nanocomplex (TDC@M/RPPTP NCs) using materials exhibiting different ROS sensitivities to combat chemoresistance through a spatiotemporally staged release of siP-gp and Dox. This system comprises mesoporous silica NPs (MSNs) that integrate with a ditellurium-crosslinked PEI shell (RPPT) and a thioketal-linked Dox prodrug (TK-Dox2) core. Exposure to low ROS levels in tumor cells triggers the rapid release of siP-gp, downregulating P-glycoprotein (P-gp) expression by >80%, thereby reversing multidrug resistance (MDR). Subsequent light irradiation generated high ROS concentrations, cleaving the thioketal linker in TK-Dox2 and releasing active Dox. This hierarchical responsive mechanism ensured MDR reversal before Dox activation, markedly increasing Dox accumulation in drug-resistant tumor cells and enhancing chemosensitivity. Consequently, a nearly complete tumor growth inhibition (98%) was achieved, providing an innovative strategy for precision nanomedicine 115. Trisulfide groups effectively clear excessive ROS within cells, conferring cellular protection and potential anti-inflammatory effects. Therefore, Wang *et al.* constructed a library of ROS-responsive trisulfide-derived LNPs (TS LNPs) for encapsulating interleukin-4 (IL4) mRNA. The TS2-IL4 LNP-mRNA induced enhanced wound healing by scavenging ROS and inducing M_2_ macrophage polarization, demonstrating considerable potential for clinical translation 116.

#### 2.2.2 GSH-responsive nanocarriers

GSH, a γ-glutamyl-cysteinyl-glycine tripeptide, is the most abundant non-protein thiol in mammalian tissues, playing a crucial role in detoxification and maintenance of cellular redox homeostasis 117. Notably, tumor cells exhibit markedly elevated intracellular GSH levels (2-10 mM), 100-1000 times higher than that in ECM (2-10 μM) and 4-10 times higher than that in normal tissues 101, 118 (Figure 4). This pronounced GSH gradient in tumors facilitates precise RNA release from stimuli-responsive nanocarriers.

As classical GSH-sensitive linkages, disulfide and diselenide bonds have been widely used in GSH-responsive drug delivery systems (Figure 5) 101, 109. Huang *et al.* designed a cation-free siRNA-PLK1 nanocapsule (T-SS(-)) featuring a disulfide-crosslinked interlayer. Elevated intracellular GSH levels triggered disulfide bond cleavage, inducing NP disassembly and achieving 90% siRNA release within 4 h. Consequently, in PC-3 xenografts, the PLK1 mRNA expression was reduced by 77% and tumor growth was suppressed by 84.3%. The platform avoids cation-related toxicity, thereby providing a safe and translatable strategy for precise cancer therapy 119. Zhang *et al.* developed a GSH-responsive nanocapsule (ApoE-MT/siPKM2 NC) for the treatment of GBM by combining siRNA-mediated inhibition of aerobic glycolysis with temozolomide (TMZ) chemotherapy. The system used a disulfide-crosslinked methacrylate-TMZ (MT) shell encapsulating siPKM2 and was further modified with apolipoprotein E (ApoE). In GSH glioma cells with high GSH levels, the systems disassembled, releasing 81.06% MT and 76.61% siRNA within 48 h, resulting in a 56.36% PKM2 knockdown. This dual-targeting strategy achieved 78.34% tumor suppression and prolonged median survival to 51 days in mice, thereby outperforming monotherapy. By coupling metabolic therapy with chemotherapy, this platform presents a promising approach to overcoming GBM chemoresistance 120.

Tetrahedral DNA nanostructures (TDNs) have emerged as a promising platform for nucleic acid delivery owing to their exceptional addressability, programmability, and high loading capacity. However, current TDN systems mainly serve as static nucleic acid carriers and lack dynamic, stimuli-responsive release elements, thereby limiting their applicability in complex biological environments. To overcome this limitation, Chen *et al.* innovatively developed GSH-responsive tetrahedral DNA-RNA nanocages (TDRNs) by precisely embedding siRNA into the DNA framework through disulfide bonds, enabling Dox and RNA co-delivery (Figure 9B). Disulfide bond cleavage triggered the efficient release of siP-gp, which synergistically suppressed the progression of MDR tumors with Dox. Their study provides a valuable reference for developing programmable TDN systems for nucleic acid-chemotherapy co-delivery 121.

Pt (IV) complexes are effectively reduced to Pt (II) in the presence of reductive substances, such as GSH, which not only restores their chemotherapeutic activity but also promotes the structural disassembly of Pt (IV)-based nanocarriers, making them promising candidates for designing stimuli-responsive drug delivery systems 101. Fan *et al.* developed a cationic polylysine-cisplatin prodrug (Pt [IV]-PLys, PP), which self-assembled with antagonist-330-3p via electrostatic interactions to form PP@miR NPs. The NPs gradually degraded in response to elevated GSH levels, enabling efficient release of ~27% of antagonist-330-3p after 6 h, along with the concurrent activation of cisplatin. Notably, in subcutaneous oral squamous cell carcinoma (OSCC) models, PP@miR NPs exhibited significant antitumor efficacy, resulting in the complete regression of 2 out of 5 tumors 122.

The incorporation of biodegradable lipids improves the tolerability of LNPs by promoting rapid metabolism while maintaining the efficacy of mRNA delivery 59. For example, incorporating disulfide bonds enables rapid RNA release and enhances transfection efficiency, making it an ideal choice for RNA-based therapeutics 123. Building on this, novel bioreducible ILs have been developed to improve the safety and efficiency of LNP delivery. These lipids, including L-PGTA 124, 2N12B 125, and 4A3-SCC-PH 126 (Figure 6), exhibit favorable degradation kinetics and enhanced biocompatibility, potentially accelerating the clinical translation of LNPs.

Despite the remarkable progress in redox-responsive nanocarriers for tumor-targeted RNA delivery, their clinical translation remains challenging. First, biocompatibility and toxicity are critical concerns. Highly reactive materials and their degradation products may exhibit potential toxicity, thereby necessitating comprehensive *in vivo* studies to evaluate their long-term safety and metabolic pathways. Second, the spatiotemporal heterogeneity of the TME poses additional challenges. The concentrations of redox-related substances in tumor tissue may be insufficient to activate nanocarriers, and the carrier-mediated response may regulate the redox levels in turn, thereby impacting therapeutic outcomes. Therefore, elucidating the underlying mechanisms and balancing the response sensitivity and cyclic stability of carriers are necessary to gain further understanding of the SAR of redox-responsive nanocarriers. Third, hypoxia, which is a hallmark of most solid tumors, impedes ROS generation, potentially alleviating the synergistic effects of redox-responsive nanocarriers combined with PDT and sonodynamic therapy (SDT). The development of hypoxia-responsive carriers, self-oxygenated nanosystems, and oxygen-independent ROS-generating technologies are expected to overcome the current dilemma. Selective enhancement of tumor oxidative stress through ROS increase and GSH depletion has recently become a promising strategy. In future, the integration of *in situ* TME monitoring technologies with dynamic RNA release strategies will be critical for facilitating the clinical translation of redox-responsive nanocarriers.

### 2.3 Enzyme-responsive nanocarriers

Enzymes are indispensable biomolecules that participate in nearly all biological processes and are essential for maintaining normal physiological functions 127. In tumor tissues, multiple enzymes, such as matrix metalloproteinase (MMP), cathepsin B (CTSB), prostate-specific antigen (PSA), hyaluronidase (HAase), and esterase are often overexpressed compared with normal tissues 14, 128. This pathological dysregulation has inspired the rational design of enzyme-responsive nanocarriers that enhance RNA delivery through enzymatic substrate cleavage (Table 3). Furthermore, the mild reaction conditions, high specificity, and favorable biocompatibility of these systems indicate their significant potential for clinical translation 129, 130.

#### 2.3.1 MMP-responsive nanocarriers

MMPs are zinc-dependent endopeptidases that degrade the ECM 141. MMPs are highly expressed in various tumors; in particular, MMP-2 and MMP-9 have been widely investigated as endogenous triggers for targeted drug delivery 142, 143. Li *et al.* developed an amphiphilic dendrimer engineered nanocarrier system (TMSP-ADENS) for the co-delivery of paclitaxel (PTX) and siVEGF. This system incorporated TME-sensitive polypeptides (TMSP), in which CPPs are masked by EGG repeat shielding domains via MMP-2/9-cleavable linkers (PVGLIG). In MMP-rich TME, the cleavage of the PVGLIG sequence induced the removal of shielding groups and exposure of CPPs, thereby significantly enhancing cellular uptake, tumor accumulation, and deep penetration. After cellular internalization, the G0-C14 dendrimer facilitates efficient endosomal escape, thereby improving the cytoplasmic delivery of both payloads. In A375 xenograft models, treatment with TMSP-ADENS/siRNA/PTX achieved a 73% reduction in VEGF mRNA expression and substantial inhibition of tumor growth without relapse 131.

Although tumor immunotherapy has made considerable clinical advancements, its efficacy remains suboptimal for many patients, highlighting the urgent need for novel therapeutic strategies. RNA-based immunotherapy has attracted substantial attention due to its versatility, high safety, low cost, and potential for personalized treatment 144. Several clinical trials are currently underway (Table 4), providing new opportunities to overcome the bottlenecks of current cancer immunotherapies. Yi *et al.* designed an MMP-2-responsive micelleplex (P^A7R^@siPD-L1) that integrated vascular normalization and PD-L1 silencing to convert "cold" tumors into "hot" tumors. The system used an MMP-2-cleavable GPLGVRG segment, allowing the tumor-specific disassembly of micelleplex and exposure of the R9 peptide to enhance cellular uptake. Meanwhile, the release of the antiangiogenic peptide (A7R) normalized the tumor vasculature, alleviating hypoxia and promoting immune cell infiltration. Under laser irradiation, the Ce6-generated ROS induced ICD and disrupted lysosomal membranes to facilitate siPD-L1 release. This multimodal immune reprogramming strategy enhanced antitumor immune responses, elicited durable immune memory, achieved complete regression in 2 out of 5 tumors, and effectively suppressed metastasis 132. Similarly, Zhang *et al.* reported an MMP-2-responsive "core-shell" nanosystem (MSL^-LY^/Pro_-siPD-L1_) to remodel the immunosuppressive TME in TNBC by simultaneously inhibiting TGF-β inhibition and silencing PD-L1. The system comprises an MMP-2-cleavable lipid shell composed of GPLGIAGQ substrates, encapsulating the TGF-β receptor inhibitor LY3200882 (LY) and the cationic protamine/siPD-L1 complex. In the MMP-2-overexpressing TME, enzymatic cleavage of the shell triggered LY release and exposed positively charged nanocore. LY suppressed TGF-β expression by 71% and promoted ECM degradation, increasing the tumor penetration depth from 28.6 to 76.2 μm in 3D spheroids and enhancing CD8^+^ T cell infiltration by 5.4-fold. The exposed nanocore facilitated siPD-L1 uptake by tumor cells and fibroblasts, reducing the PD-L1 expression to 22.8%. Furthermore, LY induced ICD in tumor cells, resulting in a 2.9-fold increase in dendritic cell (DC) maturation and a 6.2-fold enhancement in MHC II-mediated antigen presentation. This dual-blockade strategy effectively suppressed the growth, metastasis, and recurrence of TNBC, highlighting its potential as a next-generation immunotherapy platform 133.

Photothermal therapy (PTT) has been considered to be crucial for cancer treatment. However, its efficacy is limited by the upregulation of heat shock proteins (HSPs) 145. Therefore, Sun *et al.* developed an MMP-2-responsive nanoplatform (AGC/HSP-70 siRNA) by conjugating gold nanorods (AuNRs) with chitosan (CS)-siRNA complexes via an MMP-2-sensitive linker (GPLGLAG). The system was hydrolyzed by MMP-2 into smaller and positively charged CS-siRNA complexes, leading to enhanced tumor penetration, cellular uptake, and lysosomal escape. The released siRNA effectively silenced HSP-70 expression, reducing the mRNA and protein levels by 56.0% and 57.3%, respectively. This dual-action platform enhanced tumor sensitivity to PTT, achieving 61.3% tumor growth inhibition and presenting a paradigm for improved photothermal gene combinatorial therapy 134.

#### 2.3.2 Other enzyme-responsive nanocarriers

CTSB, a lysosomal cysteine protease, is highly overexpressed in various malignancies, such as breast, thyroid, liver, and colorectal cancers. Peptide-based enzyme-responsive drug delivery systems have emerged as novel intelligent platforms for an efficient RNA delivery due to their programmability, biocompatibility, and modularity. Shi *et al.* constructed a CTSB/GSH dual-responsive fluorinated peptide nanocarrier (PFC-PR) by integrating a CTSB-cleavable GFLG sequence into polyarginine (PR) and conjugating perfluorocarbon via disulfide bonds. In tumor cells, overexpressed CTSB and elevated GSH levels induce nanocarrier dissociation, facilitating siRNA release and enhancing gene-silencing efficiency, thereby confirming its potential application in anticancer gene therapy 136.

PSA, a serine protease overexpressed in prostate cancer (PCa), serves as a diagnostic biomarker and a therapeutic target 146. Prostate-specific membrane antigen (PSMA), an upregulated folate transporter in PCa, improves the uptake of folate-modified nanocarriers 147. Based on this, we developed a multifunctional liposome (AF-L) incorporating a PSA-responsive peptide (ACPP) and folate ligands for an efficient siRNA delivery. ACPP comprises PR, a PSA-sensitive linker (HSSKYQ), and shielding domains. In the PSA-rich TME, ACPP cleavage to expose PR, enhancing tumor penetration and cellular uptake. This platform achieved considerable PLK-1 silencing and effectively induced apoptosis in PCa cells, demonstrating potent therapeutic efficacy 138.

**Figure 10:**
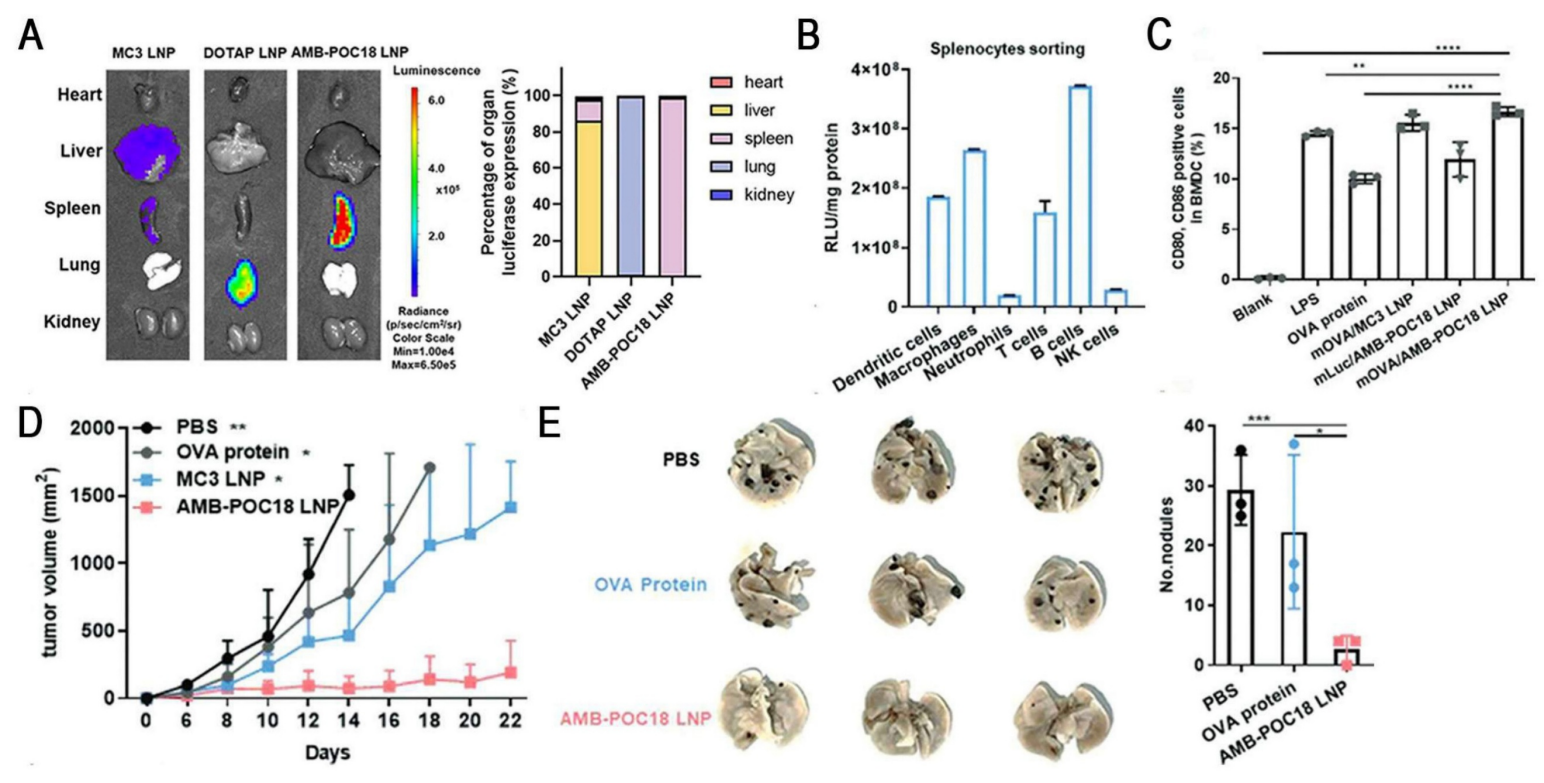
** Esterase-responsive LNPs for spleen-specific and efficient mRNA transfection to enhance cancer immunotherapy.** (A) Representative images and quantification of luminescence in major organs of MC3 LNP, DOTAP LNP, and AMB-POC18 LNP (mLuc: 0.5 mg kg^-1^). (B) Quantification of luciferase expression in splenocyte subsets 6 h after i.v. injection of mLuc/AMB-POC18 LNPs. (C) Flow cytometry analysis of CD80^+^CD86^+^ cell ratios in BM-DCs after the indicated treatments (mOVA, OVA protein, or LPS: 1 μg mL^-1^). (D) Tumor growth curves of B16-OVA tumor-bearing C57BL/6 mice in different groups. (E) Images of lung tissues and quantification of average metastatic foci. Adapted with permission from 139, copyright 2023, Wiley-VCH GmbH.

Esterase, an overexpressed enzyme in tumor cells, has attracted significant attention as a trigger for stimuli-responsive drug delivery systems 148. Zhang *et al.* constructed a library of esterase-labile lipidoids with different quaternary ammonium head groups, esterase-responsive moieties, and hydrophobic tails to address the stability-translation tradeoff in mRNA-LNP delivery (Figure 10). These lipidoids carried positive charges to ensure stable mRNA encapsulation during storage and under physiological conditions, but rapidly reversed to negative charges upon esterase-catalyzed hydrolysis, accelerating mRNA release. Orthogonally optimized AMB-POC18 LNPs (Figure 6) exhibited superior mRNA transfection efficiency of 84.9% and enhanced endo-/lysosomal escape. Moreover, they achieved spleen-specific mRNA transfection *in vivo*, particularly in antigen-presenting cells (APCs). This precise delivery promoted DC maturation and antigen presentation, thereby enhancing antitumor immunity and significantly inhibiting melanoma growth and metastasis, revealing its potential for precision RNA immunotherapy 139.

HAase, which is overexpressed in various malignant tumors, promotes tumor invasion and metastasis by degrading hyaluronic acid (HA) 149. HA, a natural anionic glycosaminoglycan, demonstrates excellent biocompatibility and tumor-targeting ability, making it a key component of smart drug delivery systems 150. Yang *et al.* developed a HAase/GSH-responsive nanoplatform (HA-LSL/siTGF-β) to elicit robust antitumor immunity in TNBC by comprehensively remodeling the TME. This system comprises a disulfide-crosslinked low-molecular-weight PEI core for siRNA compaction and a thiolated HA shell for CD44-mediated targeting of cancer-associated fibroblasts (CAFs) and tumor cells. Upon reaching acidic endo/lysosomes, the HA shell degrades to promote endo-/lysosomal escape, followed by rapid siTGF-β release triggered by GSH, thereby resulting in an 86% reduction in TGF-β expression *in vivo*. This dual-responsive platform inhibited CAF activation and depleted ECM components, improving immune cell infiltration and nanomedicine penetration. Furthermore, it reversed the immunosuppressive TME by normalizing the tumor vasculature and inhibiting EMT. Combined with anti-PD-L1, the platform significantly suppressed the growth of primary and distant tumors by 79.1% and 86.7%, respectively, and prevented tumor metastasis to the lung (less than 3 nodules). Overall, this strategy provides a promising approach for immunotherapy of stroma-rich tumors via "TME remodeling-immune activation" 135.

Enzyme-responsive NPs have attracted considerable interest for smart RNA delivery owing to their specific activation in pathological microenvironments. However, their clinical application still faces several limitations. One major limitation is tumor heterogeneity. Enzyme activity varies across patients, tumor types, and cancer stages, affecting the universality of enzyme-responsive nanocarriers as well as the consistency and predictability of therapeutic effect. Second, the limited reactivity and specificity of current enzyme-sensitive linkers may compromise the therapeutic efficacy and increase the side effects. Furthermore, the complexity of the nanocarrier design and the unclear *in vivo* mechanisms hinder their clinical translation. In future, the advancement of single-cell omics and real-time enzyme activity monitoring may address tumor heterogeneity and promote personalized RNA therapies. Moreover, AI- and DL-based models for the prediction of enzyme-substrate interaction are expected to facilitate the development of highly specific and reactive linkers, thereby accelerating the construction of next-generation smart delivery systems.

## 3. Exogenous Stimuli-Responsive Nanocarriers

Although endogenous stimuli-responsive nanocarriers provide innovative strategies for RNA delivery, the heterogeneity of the disease microenvironment hinders precise RNA release 151. By contrast, exogenous stimuli-responsive nanocarriers achieve remote, spatiotemporally controlled RNA release at lesion sites by precisely responding to external stimuli (*e.g.*, light, magnetic fields, or US), overcoming these limitations 17, 152. These systems have obvious advantages: (1) precise control over stimulus parameters (*e.g.*, intensity and location), (2) on-demand application or termination of stimuli, (3) integration of multiple technologies for multifunctional cancer theranostics, and (4) repeated or continuous stimulation for drug delivery and therapy 16. Therefore, exogenous stimuli-responsive nanocarriers have become the focus in smart nanomedicine.

### 3.1 Light-responsive nanocarriers

**Figure 11:**
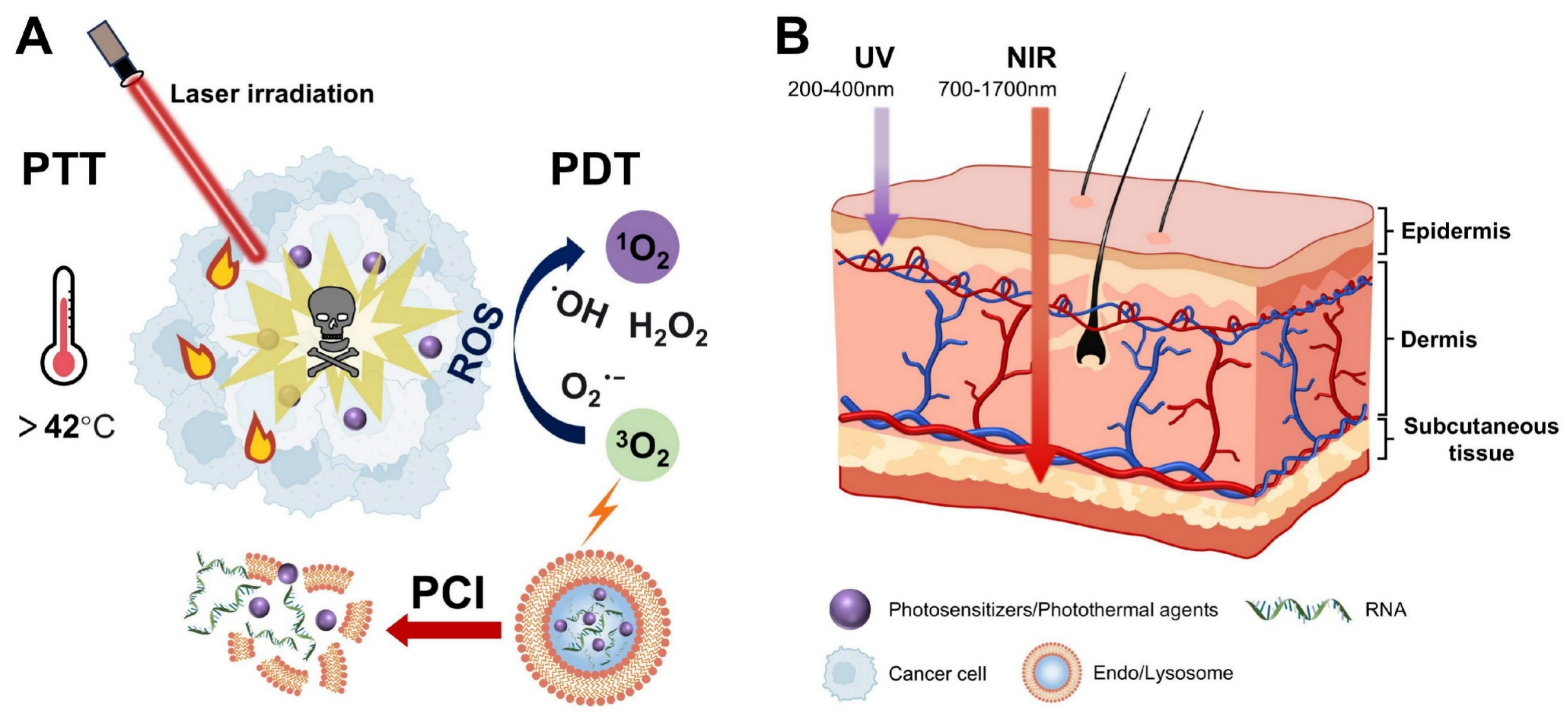
** Mechanisms and light penetration depths of phototherapies.** (A) Schematic illustration of the mechanisms of PTT, PDT, and PCI. (B) Comparison of tissue penetration depths between UV and NIR. PTT: photothermal therapy; PDT: photodynamic therapy; PCI: photochemical internalization; UV: ultraviolet; NIR: near-infrared.

Light, as an ideal external stimulus, has emerged as a promising strategy for RNA-based smart delivery owing to its noninvasiveness and precise controllability 153. Light-triggered RNA release mainly occurs through the following three mechanisms: (1) photoisomerization-driven morphological transformation (*e.g.*, azobenzene moieties), (2) degradation mediated by photocleavable linkers (*e.g.*, o-nitrobenzyl derivatives) (Figure 5), and (3) nanocarrier rupture induced by photothermal or photodynamic effects 8. In addition, light can induce tumor cell death by generating localized heat or high ROS concentrations through PTT or PDT (Figure 11A), enabling a multimodal synergistic treatment when combined with RNA therapy and showing great promise for cancer treatment 154.

Light-responsive delivery systems based on ultraviolet (UV), visible, and near-infrared (NIR) light have been developed 155. While UV demonstrates superior photolytic efficiency, its clinical application is limited by poor tissue penetration and irreversible phototoxicity 156. By contrast, NIR has lower energy but achieves deeper tissue penetration and exhibits minimal phototoxicity and high cellular compatibility, resulting in its wide application in photo-triggered RNA release strategies (Figure 11B) 151, 157. Upconversion nanoparticles (UCNPs) convert NIR into UV, integrating deep-tissue penetration with strong bond-cleavage capability, which allows for precise activation of photosensitive bonds in deeper tumors 14. Based on this, Jia *et al.* developed a light-responsive nanopolyplex (T-si/UCNP) for the co-delivery of si-PLK1 and UCNPs to achieve precise NIR-controlled siRNA release. They synthesized a UV-sensitive triblock copolymer, cRGD-PEG-PAsp (EDONB)-PPHE, containing UV-cleavable 2-nitrobenzyl ester bonds, which self-assembled with UCNPs and siRNA to form the nanopolyplex. Upon 980 nm NIR irradiation, the upconverted 365 nm UV light triggered the cleavage of 2-nitrobenzyl ester linker, thereby accelerating siRNA release. The results indicated that ˃95% of siRNA-FITC was released after two irradiation cycles. In HCT116 xenograft models, this strategy effectively reduced the PLK-1 expression and significantly inhibited tumor growth, providing a controllable and universal platform for nucleic acid delivery 158.

**Figure 12:**
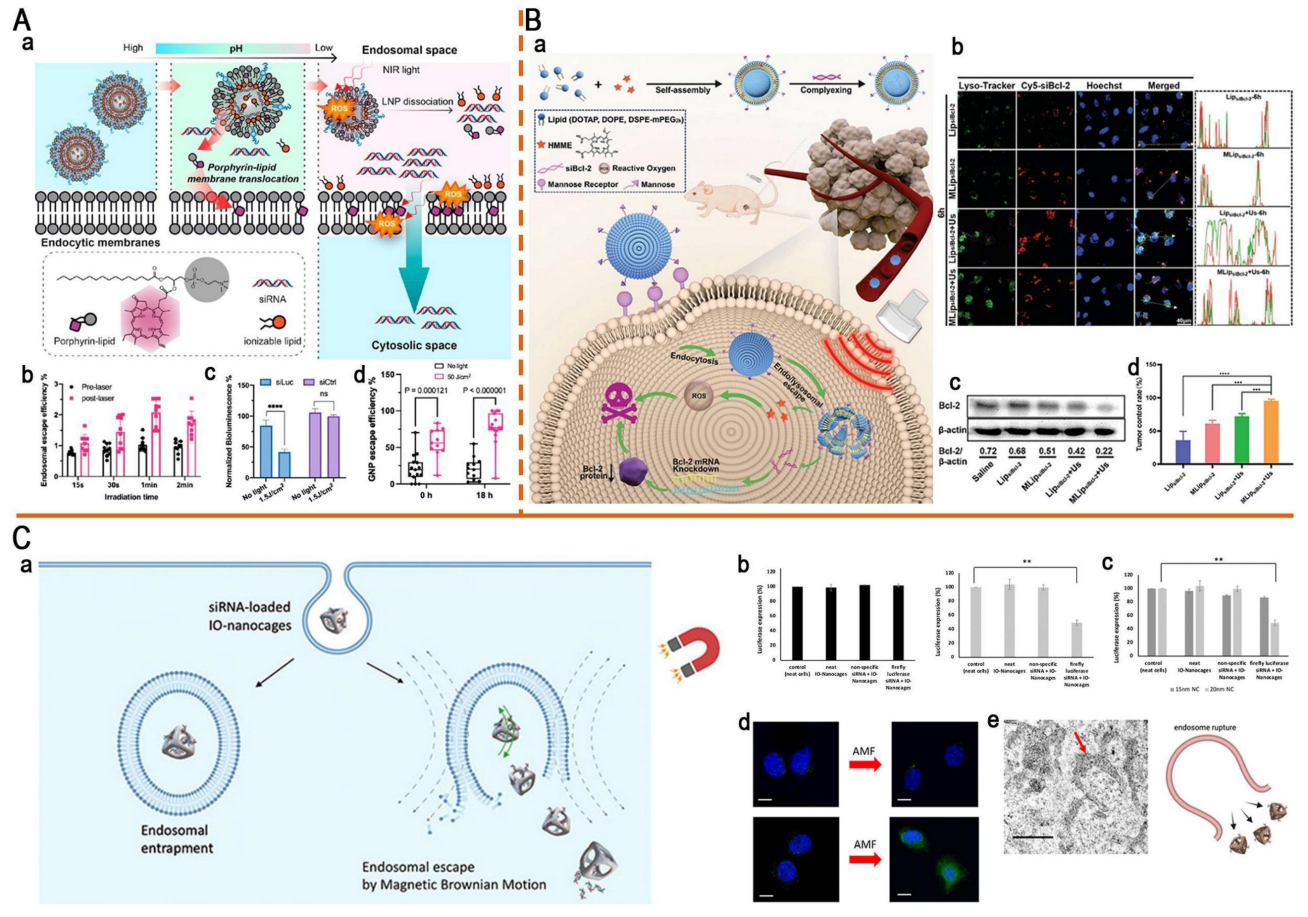
** Exogenous stimuli-responsive nano-delivery systems for RNA therapy.** (A) Light-activated siRNA endosomal release (LASER) by porphyrin-LNPs. (a) Schematic illustration of porphyrin-LNP mediated LASER approach. (b) siRNA endosomal escape efficiency pre- and post-irradiation after 6 h porphyrin-LNP incubation with cells. (c) Luciferase mRNA expression in PC3-Luc6 cells after porphyrin-LNP incubation at 10 nM siRNA dose and light treatment. (d) Quantification of GNP endosomal escape efficiency from tumors treated with and without light irradiation with dissection at the indicated time points post irradiation. Adapted with permission from 159, copyright 2023, American Chemical Society. (B) Ultrasound-sensitive targeted liposomes for gene delivery enabling synergistic treatment of hepatocellular carcinoma via SDT and gene therapies. (a) Schematic illustration of ultrasound-sensitive targeted liposomes serves as an efficient gene delivery system for the synergistic treatment of hepatocellular carcinoma with SDT and RNAi therapy. (b) CLSM images of lysosome escape of Lip_siBcl-2_ and MLip_siBcl-2_ for 6 h. Scale bars = 40 μm. (c) Bcl-2 protein expression levels of HepG2 cells after different treatments as determined by western blot assay. (d) Tumor suppression rate of different treatment groups. Adapted with permission from 178, copyright 2024, Wiley-VCH GmbH. (C) Superparamagnetic iron oxide nanocages enhance endosomal escape through magnetically induced Brownian motion. (a) Schematic illustration of Brownian motion-enhanced endosomal escape under alternating magnetic fields. (b) *In vitro* bioluminescence assay of siRNA delivery to luciferase-expressing B16-F10 cells in the left) absence and right) presence of the AMF. (c) *In vitro* bioluminescence assay of siRNA delivery to luciferase-expressing B16-F10 cells by a 15 nm IO-nanocage versus a 20 nm IO-nanocage. Brownian relaxation is more dominant for the 20 nm IO-nanocage, while Néel relaxation is more dominant for the 15 nm IO-nanocage in the AMF (f = 335 kHz). (d) Confocal images of a calcein leakage assays when B16-F10 cells were incubated with siRNA-loaded IO-nanocages of different sizes. The upper panel represents incubation with 15 nm IO-nanocages, and the lower panel represents incubation with 20 nm IO-nanocages, both in the absence left) and presence right) of AMFs. Scale bar = 20 μm. (e) TEM images of endosomes containing siRNA-loaded IO-nanocages in B16-F10 cells after the application of Aan MF (f = 335 kHz). Scale bar = 200 nm. Endosomes and IO-nanocages in cells indicated by red arrows in TEM images are illustrated to the right of each image. Adapted with permission from 183, copyright 2022, American Chemical Society.

Low endo-/lysosomal escape efficiency remains a critical challenge in RNA delivery. To address this, Mo *et al.* reported a "light-activated siRNA endosomal release" (LASER) strategy and constructed light-responsive porphyrin-LNPs (Figure 12A). They replaced 10 mol% of the helper lipid DSPC in the Onpattro formulation with porphyrin-lipid, conferring the photoactive properties while preserving the original properties of LNPs. During endocytosis, the acidic endosomal microenvironment induced porphyrin-lipid dissociation and subsequent translocation to the endosomal membranes. After a 660 nm NIR irradiation, porphyrin-lipids generated ROS, inducing LNP dissociation and endosomal membrane disruption, thereby doubling the mean endosomal escape efficiency from 1.0% to 2.1%. In addition, it markedly enhanced gene-silencing efficacy, increasing luciferase knockdown from 15% (without light) to 58% (light-treated) and decreasing the IC_50_ of porphyrin-LNP-siLuc from 8.81 to 2.15 nM. Notably, *in vivo* studies also confirmed a 2.5-3.5-fold increase in endosomal escape efficiency, providing a simple strategy to overcome RNA delivery barriers 159.

However, the tissue penetration depth of 660 nm NIR remains low (1-3 mm), which is difficult to meet the needs of deep tissue treatment. By contrast, the second near-infrared (NIR-II, 1000-1700 nm) exhibits deeper tissue penetration (5-20 mm) and lower phototoxicity, providing new opportunities for the development of light-responsive delivery systems. Li *et al.* designed a pH-activatable NIR-II dye-conjugated lipid (Cy-lipid) to enhance mRNA translation efficiency via the "stimulus-responsive photothermal-promoted endosomal escape delivery" (SPEED) strategy. In acidic endosomal microenvironments, Cy-lipid undergoes protonation, activating NIR-II absorption for an efficient light-to-heat transduction under 1064 nm laser irradiation. Subsequently, heat-induced LNP morphological changes result in a significant increase in particle size from 127.8 to 4426.8 nm, facilitating mRNA endosomal escape. Importantly, this strategy achieved a ~3-fold enhancement in *in vitro* protein expression and a 4.5-fold improvement in liver mRNA translation efficiency compared with non-irradiated controls. This study provides a promising paradigm for developing delivery systems that can efficiently deliver mRNA to deep tissues with high spatiotemporal precision and low toxicity 160.

Two-dimensional (2D) nanomaterials are highly important in disease theranostics and nanodrug delivery owing to their excellent photothermal-conversion efficiency and versatile payload capacity. PTT in combination with RNA therapy can significantly enhance antitumor effect. Yin *et al.* developed a NIR-II light-controlled CRISPR/Cas9 delivery system (silicene-Cas9) based on 2D silicene nanosheets to enhance photothermal tumor ablation through remote regulation of the TME. The system facilitates endo-/lysosomal escape and accelerates Cas9/sgRNA RNP release via the PTT triggered by NIR-II laser activation. Previous studies have shown that it effectively reprogrammed the TME by specifically knocking out the TXNDC5 gene (editing efficiency: 47.68%), thereby markedly enhancing PTT efficiency and achieving complete tumor eradication without recurrence. This intelligent nanoplatform not only addresses the limitations of PTT but also paves the way for the application of 2D materials in precision medicine 161.

Under laser irradiation, the ROS generated by PS not only directly kills tumor cells but also disrupts endo-/lysosomal membranes via the photochemical internalization (PCI) effect, thereby promoting endo-/lysosomal escape (Figure 11A) 162. Therefore, the combination of PDT and RNA therapy significantly enhances the cytoplasmic delivery of RNA via spatiotemporally controlled ROS production. Hu *et al.* developed a mitochondria-targeted nanoplatform (TPS@Ce6/miRNA NPs) that integrated PDT and RNA therapy to improve TNBC treatment. This platform used a triphenylphosphine-modified amphiphilic polymer (TPP-TPGS, TPS) to co-encapsulate Ce6 and miR-34a. After NIR irradiation, the nanocarrier induced PDT and facilitated the successful escape of miR-34a from lysosomes through the PCI effect. Furthermore, it effectively inhibited mitochondrial respiration, alleviated tumor hypoxia, and synergistically enhanced PDT efficacy through miR-34a-mediated GSH depletion. This study integrates subcellular targeting, PDT, and gene therapy, offering a promising strategy for TNBC treatment 163.

### 3.2 Ultrasound-responsive nanocarriers

US, a non-invasive mechanical wave with frequencies of >20 kHz, holds great promise for precise RNA delivery due to its capacity for deep tissue penetration, excellent spatiotemporal controllability, and inherent theranostics capabilities 164. US improves gene delivery efficiency mainly through thermal and non-thermal effects. The thermal effects arise from the conversion of acoustic energy into heat, increasing local tissue temperature. The non-thermal effects mainly include cavitation, acoustic radiation force (ARF), acoustic droplet vaporization (ADV), and SDT 165. Notably, US can directly destroy tumor cells through thermal ablation and mechanical effects, thereby remodeling the TME and enhancing immune activation 166. Given these advantages, US-responsive nanocarriers have received widespread attention. Sun *et al.* developed an innovative US-responsive nanovaccine (CM-RNA@Ce6/PLGA), encapsulating engineered cancer cell membrane proteins, mRNA, and the sonosensitizer to induce robust antitumor immunity. After subcutaneous injection, the nanovaccine efficiently accumulated in the lymph nodes and was internalized by APCs. US irradiation promoted endo-/lysosomal escape and enhanced antigen presentation, thereby activating cytotoxic T lymphocytes (CTLs) and reducing Treg infiltration. In 4T1 syngeneic mouse models, the nanovaccine significantly inhibited tumor growth and metastasis. Overall, this strategy provides a valuable reference for the development of novel personalized cancer vaccines and is expected to facilitate clinical translation 167.

The enhanced permeability and retention (EPR) effect was once regarded as the "gold standard" in nanodrug design. However, the clinical efficacy of nanomedicines is far below expectations, raising widespread skepticism regarding the validity of the EPR effect 168, 169. US-mediated cavitation has been demonstrated to enhance the EPR effect, thereby improving tumor-targeted nanodrug delivery 170. In particular, ultrasound-targeted microbubble destruction (UTMD) substantially improves gene delivery efficiency by forming transient pores in tumor blood vessel walls or cell membranes 171. Chen *et al.* designed a US-responsive theranostics nanodroplet (FH-V9302-siGLUL-NDs) to disrupt the glutamine metabolism interaction between CAFs and melanoma cells while remodeling the TME. US irradiation enhanced cellular uptake by increasing membrane permeability and simultaneously facilitated rapid payload release. This dual mechanism markedly enhanced siRNA delivery efficiency, achieving 64% GLUL knockdown in CAFs and 50% silencing in melanoma cells, thereby effectively blocking glutamine metabolism in both cells. Furthermore, the platform suppressed CAF activation, thereby promoting ECM degradation and improving nanodrug penetration while serving as a contrast-enhanced ultrasound (CEUS) imaging agent to improve tumor imaging. This integrated strategy exhibited potent antitumor efficacy, reducing tumor weight to 9.34% of controls and providing a new paradigm for the precise diagnosis and treatment of malignant tumors, such as melanoma 172.

The BBB seriously hinders the delivery of RNA to the central nervous system (CNS). Focused ultrasound (FUS) has been employed to promote BBB opening, laying the foundation for RNA therapies targeting CNS diseases and brain tumors 173. Ogawa *et al.* first reported that systemically administered mRNA-LNPs can induce foreign protein expression across the BBB after FUS-mediated BBB opening. FUS at 1.5 kW/cm^2^ induced BBB opening, resulting in a substantial increase in luciferase expression to 7.1 pg/mg protein, which was ~20-fold higher than that in a non-irradiated area without apparent bleeding or edema in mice, thereby providing a safe and minimally invasive platform for brain-targeted mRNA delivery 174. Similarly, Kwak *et al.* constructed a versatile brain-targeted delivery platform for multiple types of nucleic acids by combining FUS-mediated BBB opening with long-circulating biodegradable NPs. This platform achieves efficient and precise delivery of therapeutic nucleic acids and genome editing in the brain via a systemic route, thereby providing a safe and effective strategy for gene therapy for brain disorders 175.

Although LNPs containing short-chain PEGylated lipids (*e.g.*, DMG-PEG) can enhance transfection efficiency through rapid PEG dissociation, they also tend to interact with ApoE, resulting in an unexpected liver targeting 176. Therefore, developing a spatiotemporally controllable PEG dissociation system represents a promising strategy for precise tissue targeting and efficient RNA delivery. Chen *et al.* developed US-assisted fluorinated PEGylated LNPs (F-LNPs) for spleen-targeted mRNA delivery and *in vivo* visualization of LNPs via the liquid-to-gas transition of perfluorohexane (PFH). Upon US exposure, PFH generated microbubbles, triggering the rapid shedding of fluorinated PEG lipids and destabilization of F-LNPs, thereby promoting membrane fusion, cellular uptake, and mRNA release, ultimately enhancing the efficiency of mRNA. *In vivo* studies showed that US-assisted F-LNPs achieved a 4.0-fold increase in mRNA transfection efficiency in the spleen, with 92.8% of the bioluminescence signal specifically localized to this organ. Notably, this system efficiently transfected splenic APCs, including DCs, macrophages, and B cells, and activated robust antitumor immunity, highlighting its potential for precise mRNA vaccine delivery 177.

Similar to PDT, SDT activates sonosensitizers via US to generate ROS, which induces tumor cell death and disrupts endo-/lysosomal membranes. This approach is expected to synergize with RNA therapy to enhance antitumor effects. Wang *et al.* developed US-sensitive targeted liposomes (MLip_siBcl-2_) for the co-delivery of siBcl-2 and the sonosensitizer HMME, aiming to achieve synergistic treatment of hepatocellular carcinoma via SDT and RNA interference (RNAi) (Figure 12B). Under US stimulation, HMME-generated ROS induced tumor cell apoptosis and liposomal disruption to accelerate payload release. More importantly, it can promote RNA escape, as evidenced by the reduction in lysosomal colocalization from 58.8% to 23.6% in the MLip_siBcl-2_ + US group. This dual-action mechanism markedly enhanced Bcl-2 silencing efficiency, downregulating the Bcl-2 expression levels in tumor tissues to 0.22. The combined SDT-RNAi therapy demonstrated remarkable antitumor efficacy in Hep G2 xenograft models, achieving an 84% tumor inhibition rate (TIR), providing a clinically translatable platform for cancer combination therapy 178.

### 3.3 Magnetic-responsive nanocarriers

Compared with other exogenous stimuli, magnetic fields exhibit minimal tissue interaction and superior deep-penetration capabilities throughout the body, making them one of the most effective triggering strategies for drug delivery systems 151. Magnetic nanoparticles (MNPs) enable selective tumor targeting under external magnetic field guidance, markedly enhancing RNA accumulation at tumor sites 179. Among them, superparamagnetic iron oxide nanoparticles (SPIONs) demonstrate magnetism only in the presence of magnetic fields and lose this property during their absence 180. This dynamically controllable magnetic behavior makes it an ideal RNA delivery carrier. Bao *et al.* developed superparamagnetic Fe_3_O_4_-based dual-shell MSNs (FLSNs) for efficient siPLK1 delivery into breast cancer cells to induce apoptosis. The inner dense amorphous silica layer (~30 nm) prevents oxidation of the magnetic Fe_3_O_4_ core, whereas the outer large-pore (6-50 nm) mesoporous silica shell enables effective siRNA loading and protection. Magnetic field guidance significantly enhances cellular uptake in MDA-MB-231 cells, resulting in a considerable reduction in cell viability. This "dual-shell protection with magnetic targeting" strategy provides a paradigm for the development of precision gene therapy 181.

Notably, the surface functionalization of MNPs is expected to lead to the construction of a multifunctional nanoplatform that integrates gene therapy, magnetic hyperthermia (MHT), and magnetic resonance imaging (MRI). Chen *et al.* reported a zinc-doped iron oxide octahedral multifunctional nanoplatform (ZIPP-Apt:DOX/siHSPs) for precise tumor theranostics using a multimodal synergistic strategy. The platform employed AS1411 aptamer-mediated active targeting and the "proton sponge effect" of PAMAM dendrimers to enhance the intracellular delivery of siRNA and DOX. The superparamagnetic core exhibited excellent magnetic-to-thermal conversion efficiency and supported NIR/MR dual-modality imaging monitoring. By simultaneously silencing HSP70 and HSP90, the system sensitized tumors to MHT and chemotherapy, leading to notable 4T1 tumor suppression, with complete regression in 2 out of 5 tumors. This work provides valuable insights into the development of integrated theranostics nanoplatforms, leveraging the synergistic effects of MHT, chemotherapy, and RNAi, combined with real-time imaging 182.

In biomedical fields, SPION-driven hyperthermia under alternating magnetic fields (AMFs) has been extensively investigated. However, the potential of magnetically induced Brownian motion for improving RNA delivery remains largely unexplored. Kang *et al.* designed cage-shaped SPIONs (IO-nanocages) for an efficient siRNA delivery via Brownian motion. This motion induced endosomal membrane disruption, thereby facilitating endosomal escape and cytoplasmic siRNA release (Figure 12C). The study reported that the Brownian-dominant IO nanocage (20 nm) achieved a delivery efficiency of 51%, which is 5 times higher than that of the Néel-dominant IO nanocage (15 nm) 183. They further explored the delivery of mGluR5 siRNA using IO-nanocages under AMFs to inhibit the proliferation of osteosarcoma cells. This system effectively reduced mGluR5 mRNA expression in human osteosarcoma cells (LM7) and mouse model cells (OS482) by 74.5% and 95.4%, respectively, markedly inhibiting cell proliferation. Overall, this magnetically driven IO-nanocage platform represents a promising approach for gene therapy in future clinical applications 184.

Exogenous stimuli-responsive nanocarriers hold great promise for precision cancer therapy, but their clinical translation faces several critical challenges. First, biosafety concerns must be urgently addressed as some non-biodegradable materials potentially inducing accumulation toxicity in vital organs, thereby limiting their long-term application. Therefore, it is necessary to develop materials with higher biocompatibility and degradability and to establish a systematic and standardized safety evaluation framework to support clinical translation. Another obstacle is the dependence on specialized equipment and constraints in application scenarios. Current stimulation devices (*e.g.*, magnetic field generators and high-intensity US systems) are often bulky and expensive, severely hindering their application in primary care and home settings. The integration of wearable devices (*e.g.*, flexible ultrasound patches and flexible magnetic field sensors) with intelligent feedback systems facilitates the creation of portable diagnosis and treatment platforms. Such systems are expected to achieve automated, continuous, and intelligent theranostics, thereby significantly reducing treatment costs and enhancing therapeutic flexibility. In addition, repeated stimulation raises concerns about tissue tolerance and patient compliance as high-intensity US may cause localized tissue damage and phototherapy may induce non-target heating, thereby compromising treatment sustainability. In future, the development of multimodal synergistic stimulation strategies or stimuli-memory-responsive nanocarriers could reduce the required stimulation intensity and frequency. Meanwhile, the integration of wearable devices and low-energy frequency-responsive nanocarriers is expected to achieve safe, home-based diagnosis and treatment, thereby facilitating broader clinical translation.

## 4. Other-Responsive Nanocarriers

**Figure 13:**
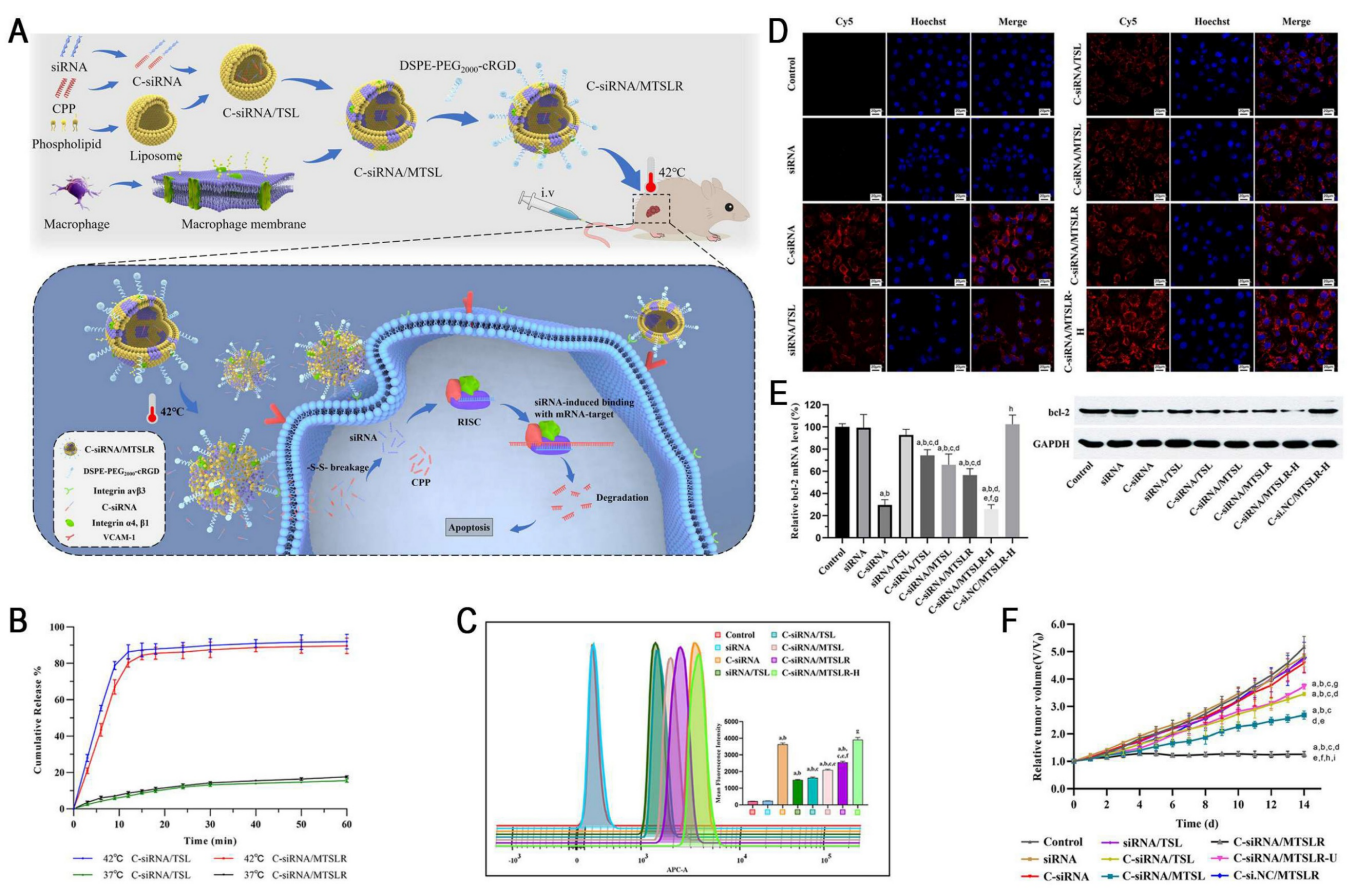
** Temperature-responsive macrophage membrane- and cRGD-functionalized liposomes combined with CPPs to realize precise siRNA delivery into tumor cells.** (A) Schematic illustration of preparation and proposed illustration of C-siRNA/MTSLR. (B) Temperature-triggered release profiles of siRNA from C-siRNA/MTSLR and C-siRNA/TSL at 37°C and 42°C, respectively. (C) Cellular uptake evaluation of various nanoparticles into HepG2 cells by FCM. (D) Cellular uptake evaluation of various nanoparticles into HepG2 cells by CLSM. Hoechst 33258 for nuclear staining (blue) and cy5 fluorescence (red) were recorded. (E) The level of Bcl-2 mRNA and protein expression in HepG2 cells treated with various samples. (F) Relative tumor volume changes in HepG2 tumor-bearing mice. (a) p < 0.05 versus Control; (b) p < 0.05 versus free siRNA; (c) p < 0.05 versus C-siRNA; (d) p < 0.05 versus siRNA/TSL; (e) p < 0.05 versus C-siRNA/TSL; (f) p < 0.05 versus C-siRNA/MTSL; (g) p < 0.05 versus C-siRNA/MTSLR; (h) p < 0.05 versus C-siRNA/MTSLR-H/U; (i) p < 0.05 versus C-si.NC/MTSLR. Note: the samples in these experiments were treated with heat except for those marked with "H" or "U". Adapted with permission from 185, an open access article under a CC-BY 4.0 license. Copyright 2021, The Authors.

Temperature-responsive nanocarriers have been widely used to achieve controlled RNA delivery. When the temperature exceeds the phase transition temperature (Tm), the phospholipids in thermosensitive liposomes (TSLs) undergo gel-to-liquid crystalline phase transition, significantly increasing membrane fluidity and permeability, thereby triggering RNA release. Nai *et al.* developed a TSL-based multifunctional delivery system (C-siRNA/MTSLR) by incorporating the macrophage membrane, tumor-targeting cRGD peptide, and CPP-siRNA conjugate to achieve precise siRNA delivery to tumor cells (Figure 13). The experimental results indicated that localized hyperthermia at 42°C in tumor regions induced the rapid release of 80% siRNA within 10 min. Subsequently, CPP-mediated potent membrane penetration enhanced the cellular uptake of siBcl-2 in HepG2 cells, leading to the substantial downregulation of Bcl-2 levels, effectively suppressing tumor progression 185.

Electroporation enhances membrane permeability and facilitates the entry of nucleic acid by creating pores in the cell membrane through the application of high-intensity electric fields. While it demonstrates high transfection efficiency, its clinical application is limited by cytotoxicity and uneven transfection. Therefore, Liu *et al.* developed a thin-film-based nanochannel electro-injection (NEI) technique for the safe and efficient delivery of various nucleic acid molecules into DCs. By localizing the electric field at the cell membrane surface using nanochannels with ~200 nm diameter, NEI required only a low voltage (<30 V) to induce transient membrane perforation, thereby markedly reducing cytotoxicity (viability >85%). Moreover, the uniform distribution of nanochannels ensured homogeneous transfection and drove nucleic acid directly into the cytoplasm via the electrophoretic effect, achieving a delivery efficiency of >70% for plasmid DNA (pDNA), mRNA, and circular RNA (circRNA) in DC2.4 cells. Notably, NEI successfully delivered circRNA into difficult-to-transfect primary bone marrow-derived DCs (BMDCs) with 68.3% efficiency without inducing BMDC maturation, thereby retaining their ability to activate T lymphocytes, highlighting its promising potential for the development of DC-based cancer vaccines 186.

Mechanical oscillation can accelerate the motion of NPs in a medium, thereby increasing their kinetic energy and potentially enhancing endosomal escape. To address the limited endosomal escape efficiency of LNPs, Chen *et al.* designed an electromagnetic shaker to evaluate the potential of mechanical oscillation for promoting endosomal escape. The study showed that the application of a 65 Hz mechanical oscillation decreased the Pearson's correlation coefficient (PCC) from 0.60 to 0.46, indicating enhanced endosomal escape. More importantly, this strategy improved the efficiency of mRNA transfection while maintaining high cell viability (99.3%), leading to an increase in eGFP mRNA fluorescence intensity by 67.9% and 243% in FNE and A549 cells, respectively. In addition, the mechanical parameters used were generally considered safe for the human body, highlighting the potential for clinical translation. In summary, the study presents a low-cost and highly translatable strategy for promoting endosomal escape, with broad application prospects in biomedical fields, such as RNA delivery 187.

## 5. Dual- or Multi-Responsive Nanocarriers

Single stimulus-responsive nanocarriers often exhibit suboptimal selectivity, increasing the risk of off-target release in healthy tissues. By contrast, dual- or multi-responsive nanocarriers can integrate various internal and external stimuli to achieve a more efficient and precise gene delivery, thereby enhancing antitumor efficacy. Dual pH-responsive nanocarriers combine pH-labile bond cleavage and chemical group protonation/deprotonation mechanisms to achieve hierarchically responsive release across extracellular and intracellular environments, significantly improving RNA delivery 188, 189. In our previous study, we developed a multi-stage pH-responsive liposome (D-L/si-DTX) for the co-delivery of siPLK-1 and docetaxel (DTX) to achieve synergistic cancer therapy. This system incorporated a pH-responsive peptide (DPRP) comprising a PR cell-penetrating domain, a polyanionic shielding domain, and an acid-labile imine linker. Once the acidic TME was reached, imine bond cleavage triggered the detachment of the shielding domain, exposing the cationic PR, which significantly enhanced tumor penetration, and cellular uptake subsequently facilitated endo-/lysosomal escape. Furthermore, stearylated-octahistidine (SA-H8) electrostatically compressed siRNA in acidic endosomes. After entry into the cytoplasm, histidine deprotonation induced complex dissociation, enabling efficient siRNA release and robust PLK-1 silencing. This dual pH-responsive platform overcomes the key barriers in siRNA delivery, including extracellular penetration, intracellular trafficking, and cytoplasmic release, while integrating chemo-gene synergy for an enhanced antitumor efficacy without detectable systemic toxicity 190.

**Figure 14:**
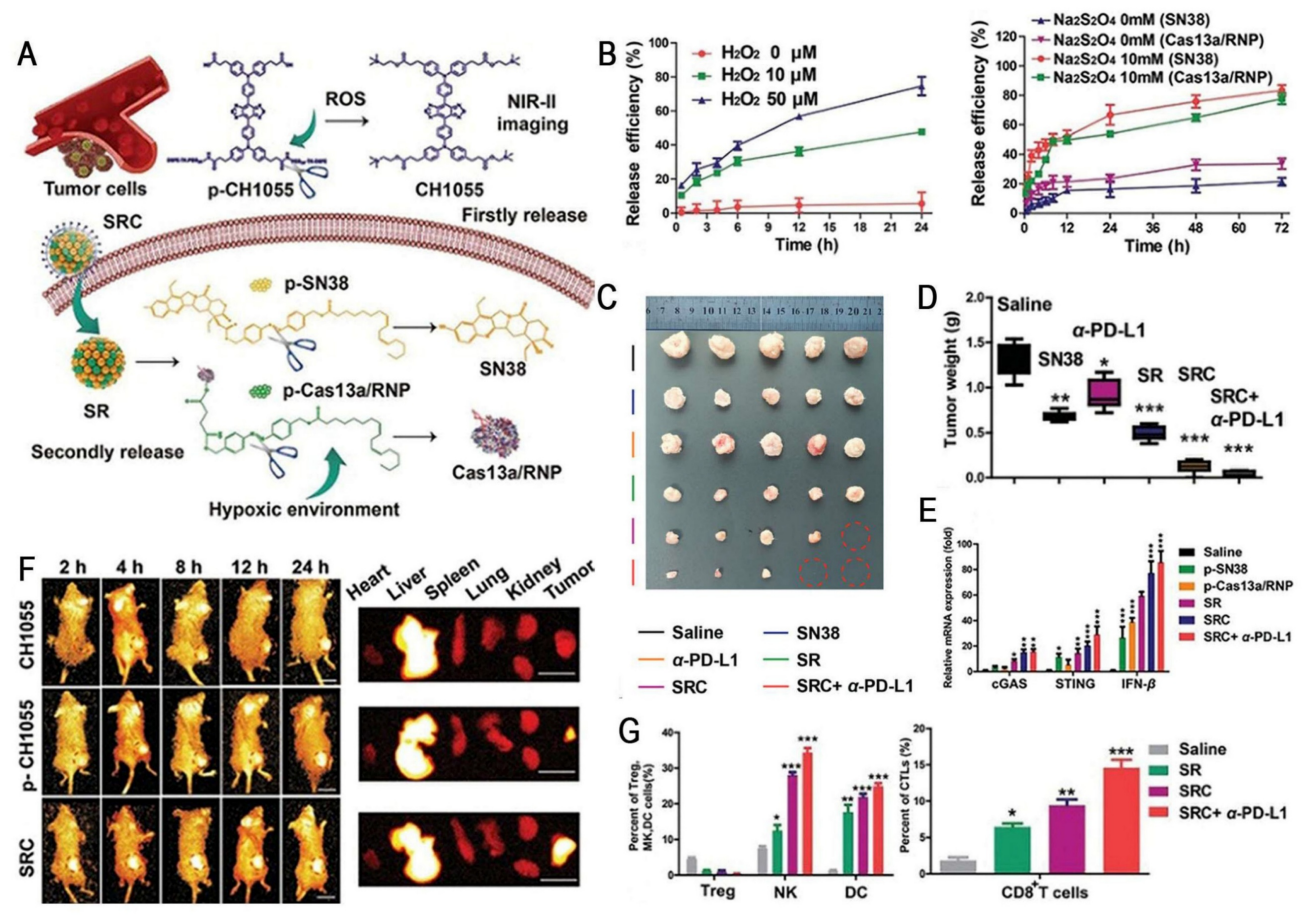
** ROS/Hypoxia-responsive cascade self-uncloaking nanoassembly (SRC) for precise CRISPR/Cas13a RNP delivery.** (A) Schematic illustration of cascade self-uncloaking and payload release of SRC. (B) left) Release profiles of CH1055 with different concentrations of H_2_O_2_. right) Release profiles of SN38 and Cas13a/RNP under hypoxic environment. (C) tumor images and (D) tumor weight after treatment with different formulations, respectively. (E) The activation of the cGAS-STING pathway was evaluated by the mRNA expression. (F) The NIR-II tumor fluorescent images of mice after intravenous injection at 2, 4, 8, 12, and 24 h, and the fluorescence images of major organs and tumors after the sacrifice of mice. Scale bar = 2 cm. (G) The quantitation of the percent of corresponding cells. Adapted with permission from 194, copyright 2023, Wiley-VCH GmbH.

CRISPR-Cas13a, a powerful gene editing tool, shows great promise for gene therapy in malignant tumors. Unlike Cas9, which targets DNA, Cas13a specifically targets and cleaves single-stranded RNA. Direct delivery of Cas13a/crRNA ribonucleoprotein complexes (Cas13a RNPs) has attracted considerable attention due to their high efficiency, reduced off-target effects, and avoidance of integration-related risks 191. Unfortunately, the broader application of Cas13a is restricted by the lack of effective delivery platforms and the need for precise tumor targeting 192, 193. Therefore, Zhang *et al.* developed a ROS/hypoxia cascade self-uncloaking nanoassembly (SRC) based on SN38 and TIM3-targeting CRISPR/Cas13a dual prodrugs to achieve precise CRISPR/Cas13a delivery (Figure 14). The system features an outer TK bond-containing probe (p-CH1055), which undergoes ROS-responsive cleavage in the TME, thereby releasing CH1055 for NIR-II fluorescence imaging and exposing the SR nanoparticle core. Subsequently, azobenzene-conjugated SN38 and Cas13a/RNP prodrugs are cleaved under intracellular hypoxia, facilitating targeted and controlled release of both therapeutics. This cascade-responsive mechanism synergizes chemotherapy, gene editing, and immune checkpoint blockade therapy, resulting in a 10-fold enhancement in tumor regression through amplified antitumor immunity. In general, this strategy validates the feasibility of CRISPR/Cas13a-based cancer therapy and provides an efficient, clinically translatable platform for an integrated diagnosis and treatment in solid tumors 194.

**Figure 15:**
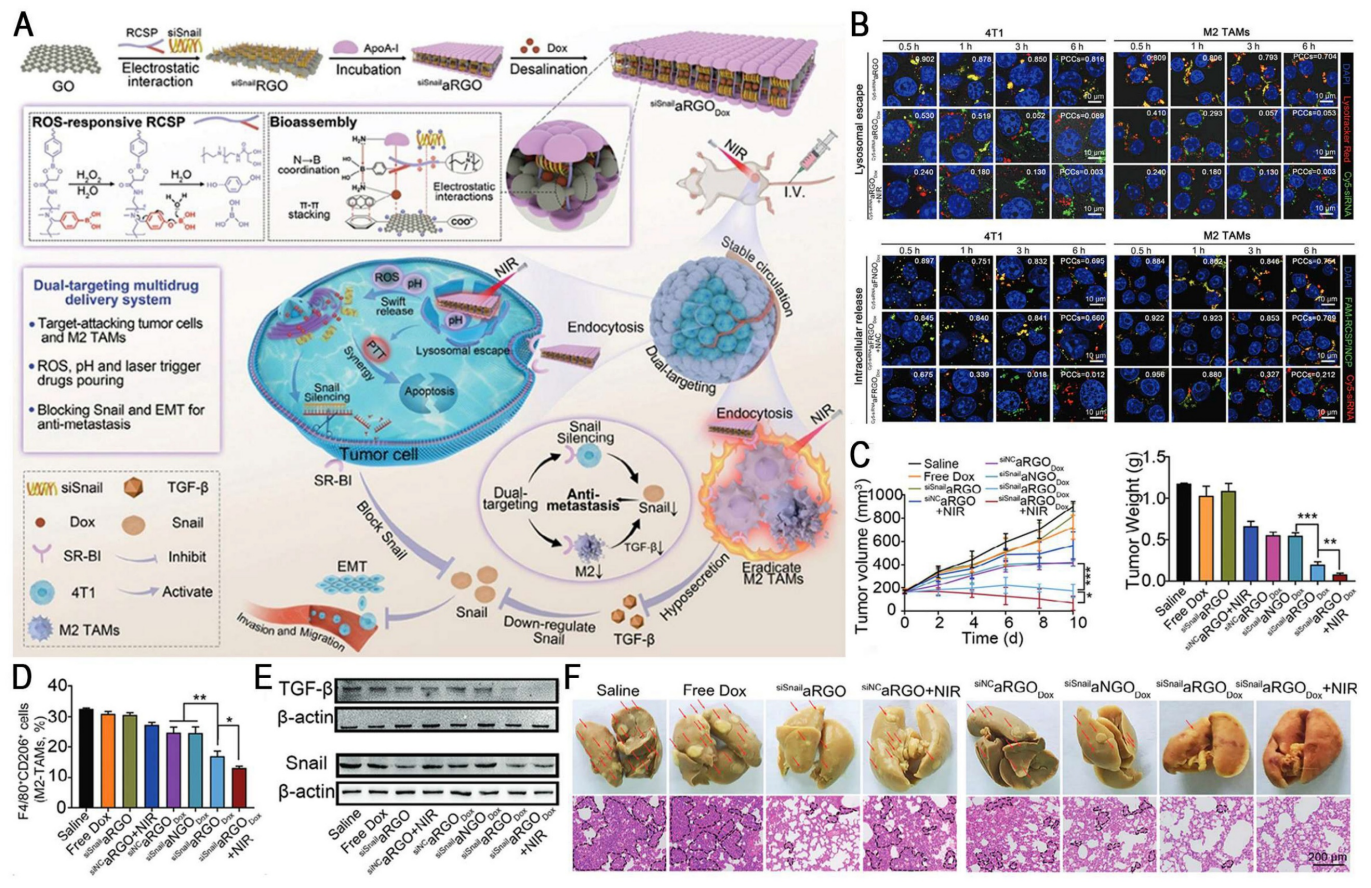
** A ROS/pH/NIR triple-responsive nanoassembly synergistically targets tumor cells and M_2_ macrophages to achieve complete metastasis inhibition.** (A) Schematic illustration of preparation process and pharmacodynamic mechanism of triple-responsive hybrid nanoparticles (^siSnail^aRGO_Dox_). (B) upper) Lysosome escape of ^Cy5-siRNA^aRGO, ^Cy5-siRNA^aRGO_Dox_ with or without laser irradiation in 4T1 cells and M_2_-TAMs. lower) Intracellular release of Cy5-labeled siRNA from ^Cy5-siRNA^aFRGO_Dox_, ^Cy5-siRNA^aFNGO_Dox_, and ^Cy5-siRNA^aFRGO_Dox_ with NAC pre-incubation in 4T1 cells and M_2_-TAMs. (C) Tumor growth curves and tumor weights in 4T1 tumor-bearing mice after treatment. (D) Quantitative analysis of M_2_ TAMs (F4/80⁺CD206⁺ cells) in tumor tissues after treatments using flow cytometry. (E) The expression of TGF-β and Snail in tumor tissues collected from the treated groups. (F) Images and H&E staining of lungs collected from different groups. Adapted with permission from 195, copyright 2024, Wiley-VCH GmbH.

Compared with single- and dual-responsive nanocarriers, multi-responsive nanocarriers enable a more specific RNA-targeted delivery, markedly reducing off-target toxicity and providing a promising strategy for a safe and effective cancer therapy. Wang *et al.* constructed a ROS/pH/NIR triple-responsive nanoassembly (^siSnail^aRGO_Dox_) by combining PBA-crosslinked short-chain polyquaternium (RCSP) with a graphene oxide (GO) scaffold, which was further modified with apolipoprotein A-I (apoA-I) for the dual-targeted modulation of tumor cells and M_2_ TAMs (Figure 15). The triple-responsive mechanism synergistically promoted DOX/siRNA release through (1) protonation of amino groups in acidic lysosomes, disrupting N:→B coordination and π-π stacking; (2) ROS-triggered PBA cleavage, eliminating the positive charge of RCSP; and (3) NIR-induced photothermal destruction of π-π stacking. ^Cy5-siRNA^aRGO_Dox_ NPs could quickly escape from lysosomes under the synergetic proton sponge effects of coordinated Dox and photothermal effect of GO. The experimental results indicated that apoA-I-mediated targeting enhanced Cy5-siRNA accumulation in M_2_ TAMs by 3.6-fold compared with the non-targeted group, with similarly high uptake in 4T1 tumor cells. The combined therapy induced apoptosis in 4T1 cells and M_2_ TAMs at rates of 59.9% and 68.8%, respectively (IC_50_ as low as 0.51 and 0.49 μg/mL), while exhibiting significantly lower cytotoxicity toward M_1_ TAMs and HUVEC cells (IC_50_, 4.11 and 6.34 μg/mL, respectively). In orthotopic 4T1 breast cancer models, this platform effectively blocked the Snail/TGF-β signaling pathway and reversed EMT by silencing Snail expression and eliminating M_2_ TAMs, ultimately achieving a TIR of 93.3% with no lung metastasis. This study is the first to integrate triple-responsive payload release mechanism, dual-targeting strategy, and multimodal therapy, providing a novel paradigm for the treatment of metastatic cancer 195.

Self-amplifying RNA (saRNA) has emerged as a next-generation platform for RNA therapy. Owing to its capacity for self-replication within the host cytoplasm, saRNA enables high-level and sustained protein expression at low doses, making it particularly suitable for the development of vaccines. The world's first approved saRNA vaccine, ARCT-154 (5 μg), has been reported to elicit a stronger immune response than BNT162b2 (30 μg) mRNA vaccine, highlighting its notable clinical potential 196. Recently, the saRNA-LNP platform has been increasingly explored in the context of cancer immunotherapy. JCXH-211 (NCT05727839), the first saRNA therapeutic encoding human IL-12 to enter clinical trials, has demonstrated excellent safety and significant antitumor activities, including abscopal effect. However, similar to other RNAs, saRNA requires a safe and efficient delivery system to maximize therapeutic efficacy while minimizing toxicity 197. Bioreducible pABOL polymers degrade in response to GSH, facilitating efficient RNA release and demonstrating low cytotoxicity 198. Moving forward, intelligent delivery strategies that incorporate dual- or multi-stimuli responsiveness are expected to further enhance the precision of saRNA delivery and expand its applications in tumor immunotherapy, infectious diseases, and genetic disorders. Although this field is still in the early stages, we believe that it will become a pivotal breakthrough in saRNA delivery.

At present, dual- and multi-responsive nanocarriers offer significant advantages, such as enhancing the precision of RNA delivery, minimizing off-target toxicity, and enabling multimodal synergistic therapies. However, the poor stability of complex carrier structures, challenges in the compatibility of multiple responsive modules, and the high cost of large-scale production limit their practical application. In future, the development of novel smart materials and AI-driven precision design strategies is expected to facilitate the real leap of dual- and multi-responsive delivery systems from laboratory to clinic.

## 6. Conclusions and Perspectives

RNA-based therapy has attracted considerable attention in cancer treatment due to its high specificity and programmability. However, its clinical application is limited by several challenges, including rapid degradation by nucleases, poor cellular uptake, limited endosomal escape, and potential immunogenicity and off-target toxicity. Although stimuli-responsive nanocarriers have significantly enhanced the accuracy and efficiency of RNA delivery, several critical obstacles must be overcome to fully realize their clinical potential:

1. The inherent complexity of the physiological environment, coupled with the pronounced heterogeneity of tumors, presents substantial challenges in achieving the precise delivery of endogenous stimuli-responsive RNA nanocarriers. However, current animal models fail to fully recapitulate the heterogeneity of human tumors, thereby limiting their predictive value for *in vivo* delivery performance and impeding clinical translation.

Building a synergistic framework of "AI-driven predictive modeling + organoid simulation" has great potential for overcoming these challenges. In particular, the integration of multimodal biosensors with AI-powered real-time monitoring systems enables dynamic analysis of *in vivo* distribution, metabolism, microenvironmental signal fluctuations, and delivery performance. Furthermore, DL algorithms can further optimize exogenous stimulus parameters to support precision medicine. Meanwhile, combining big data analysis technologies such as single-cell sequencing, spatial transcriptomics, and proteomics, to construct patient-derived organoid or organ-on-a-chip evaluation models enables a more accurate simulation of the human TME. These strategies demonstrate superior predictive value and notable potential for clinical translation in preclinical studies, which are expected to accelerate the development of personalized cancer treatments.

2. The long-term safety and biocompatibility of nanocarriers are fundamental prerequisites for their clinical translation. The toxicity of NPs is related to their physicochemical properties. Smaller NPs have been reported to exhibit higher toxicity, while positively charged nanomaterials (*e.g.*, PEI, cationic lipids) can damage cell membranes to induce cytotoxicity. Metallic NPs (*e.g.*, Ag, ZnO) can induce oxidative stress, leading to lipid, protein, and DNA damage. In addition, off-target toxicity and toxic elements (Te) further limit their clinical application. The immunogenicity of NPs depends on their composition. For example, ionizable lipids have adjuvant properties and can activate immune responses, PEG can induce anti-PEG antibodies upon repeated administration, leading to accelerated blood clearance (ABC) and potentially triggering hypersensitivity reactions. Furthermore, certain NPs elicit robust inflammatory responses by activating proinflammatory signaling pathways (*e.g.*, NF-κB, MAPK). Intravenously injected NPs tend to accumulate in the liver and spleen, while non-biodegradable materials (*e.g.*, gold, silica) exhibit poor clearance, resulting in long-term toxicity. The administration route is also a key determinant of nanotoxicity, inhalation may cause lung inflammation or fibrosis, whereas oral delivery can cause irritation in the gastrointestinal tract.

The following strategies are expected to address the abovementioned problems. First, regulating the physicochemical properties of NPs (*e.g.*, controlling size, optimizing charge) and modifying them with targeting ligands can help reduce toxicity to normal tissues. As regards the toxicity of Te-based materials, recent studies suggest that organotellurium compounds exhibit lower toxicity, indicating that structural modification may facilitate their broader application. Second, replacing PEG with novel biocompatible materials (*e.g.*, polybetaine, polysarcosine, and polycarbonate) or optimizing molecular topology (*e.g.*, branching) may decrease antibody recognition, thereby reducing immunogenicity. Additionally, incorporating degradable linkages (*e.g.*, ester, amide, and disulfide bonds) into the materials or developing enzymatically degradable materials can avoid the risk of organ accumulation. Meanwhile, adjusting surface properties may facilitate hepatobiliary or renal clearance, further reducing long-term retention in organs. Finally, rational selection of administration routes and control of dosage and frequency can improve overall safety. In summary, these approaches support the rational design of safer nanocarriers for clinical translation.

3. The clinical application of stimuli-responsive RNA nanocarriers is challenged by large-scale production and high manufacturing costs. Their complex structures result in considerable batch-to-batch variability, while the limited scalability of conventional fabrication methods severely hampers clinical translation. Moreover, high production costs and poor storage stability further impede their industrialization. To overcome these challenges, future advancements in the following two key areas are warranted: innovation in manufacturing technologies and AI-assisted design.

In terms of manufacturing, microfluidic high-throughput manufacturing technologies enable large-scale, automated production of nanomedicines by precisely regulating fluid dynamic parameters and microchannel architectures. NPs produced using this approach exhibit high batch-to-batch reproducibility, uniform morphology, excellent stability, and high encapsulation efficiency. Furthermore, this technology demonstrates strong scalability and flexibility, meeting the developmental needs of diverse delivery systems (*e.g.*, LNPs, lipid-polymer hybrid systems, hydrogels, and microparticles), thereby demonstrating broad industrial application prospects. In terms of intelligent design, ML-based virtual screening platforms can systematically analyze high-throughput datasets (*e.g.*, SAR, process parameters, and quality control indicators), thereby constructing full-chain accurate predictive models of "molecular design-process optimization-quality control". This integrative approach has the potential to facilitate the rational design of high-performance carriers, shorten research and development timelines, and mitigate trial-and-error costs, ultimately accelerating the discovery of RNA delivery systems.

In summary, although stimuli-responsive nanocarriers have significant advantages for RNA delivery, extensive research and rigorous validation are still warranted to support the development of safe, efficient, and personalized precision therapies.

## Figures and Tables

**Table 1:** Comparison of stimuli-responsive nanocarriers and traditional nanocarriers for RNA therapeutics.

Nanocarriers	Examples	Advantages	Limitations
Viral vectors	Adenovirus; AAV; Retrovirus; Lentivirus;	Excellent transfection efficiency; Sustained gene expression; Enhanced targeting specificity;	Potential immunogenicity; Complex and costly production; Limited RNA loading efficiency; Risk of insertional mutagenesis;
Lipid-based	Liposomes; SLNs; LNE;	RNA payload flexibility; Good biocompatibility; Suitable for large-scale production; High standardization;	Non-specific liver targeting; Storage and stability limitations; Potential to induce immune response; "ABC" phenomenon;
Polymer-based	Dendrimers; Micelles; Polymersomes;	Good biocompatibility and biodegradability; Controllable characteristics; Easy modification; Customizable structure;	Risk of particle aggregation; Potential cytotoxicity; Non-degradability of certain polymers; Synthetic complexity;
Inorganic NPs	AuNPs; MSNs; IONPs; QDs; CNTs;	Unique physicochemical properties; Integrated diagnosis and treatment; Easy surface modification; Controllable size, structure and shape;	Non-biodegradability; Long-term accumulation toxicity; Poor stability and biocompatibility; Potential cytotoxicity;
Protein-based	ADC; Protein cage; RBP; Poly peptides;	Strong targeting; Good biocompatibility; Efficient cellular internalization; Easy synthesis; Low cytotoxicity;	Poor stability and short half-life; Limited RNA loading efficiency; Potential immunogenicity; Complex and costly production;
Bio-inspired vehicles	Exosomes; Evs; Cell membrane-coated NPs;	Excellent biocompatibility and targeting ability; Strong cross-biological barrier capability; Extended circulation time; Low immunogenicity;	Complex and costly production; Standardization and loading challenges; Potential biosafety concerns; Stability issues;
Stimuli-responsive	Endogenous stimulus; Exogenous stimulus;	Superior RNA delivery efficiency; Spatiotemporally controlled RNA release; Enhanced targeting specificity and tissue penetration; Integrated diagnosis and treatment; High safety; Potential for personalized medicine;	Complex design and limited stability; Difficult to large-scale production and clinical translation; Safety concerns related to exogenous stimuli; Tumor heterogeneity challenges; Lack of standardized protocols;

ABC: accelerated blood clearance; ADC: antibody-drug conjugate; AAV: adeno-associated virus; AuNPs: gold nanoparticles; CNTs: carbon nanotubes; Evs: extracellular vesicles; IONPs: iron oxide nanoparticles; LNEs: lipid nanoemulsions; MSNs: mesoporous silica nanoparticles; NPs: nanoparticles; QDs: quantum dots; RBP: RNA-binding protein; SLNs: solid lipid nanoparticles.

**Table 2:** Stimuli-responsive RNA-loaded nanocarriers in cancer therapy.

Type	Stimuli	Nanocarriers	RNA	Descriptions	Ref.
Endogenous	pH	Biomimetic NPs	siRNA	· In acidic endosomes, CA hydrolysis induces NP charge conversion, disrupting the RBCm and facilitating endosomal escape; · The carrier shows a t_1/2_ of 1.6 h, 64% PLK1 mRNA downregulation *in vitro* and 82.4% TIR in GBM;	19
		SLNs	miRNA	· In the acidic TME, imine bond hydrolysis induces PEG detachment and targeting peptide exposure, enhancing cellular uptake; · The carrier achieves 87.19% miRNA-200 encapsulation, ~2-fold higher transfection efficiency than Lipo3000, and ~60% reduction in SAS cell activity;	20
		LNPs	siRNA	·In acidic endosomes, protonation of HCQ enhances endosomal escape by blocking autophagosome-lysosome fusion and increasing endosomal osmotic pressure; · The carrier achieves 87.25% siRNA encapsulation, >75% CDK4/6 silencing *in vivo*, and 61.89% TIR in BCa;	21
		Polymeric NPs	siRNA	· In acidic endosomes, protonation of PDPA induces NP disassembly, facilitating endosomal escape; · The carrier achieves ~85% siRNA encapsulation, ~60% release within 12 h, ~80% ATP6 downregulation *in vitro*, and ~80% TIR in BCa;	22
	Redox	Complex NPs	siRNA or RNP	· ROS-triggered PBAE cleavage induces PEG detachment, restoring liposomal fusion capability, and enhancing cytoplasmic delivery of siRNA or CRISPR-Cas9 RNP; · The carrier enhances endosomal escape by ~3-fold and downregulates MDK mRNA by ~75% in LN229R cells;	23
		LNPs	mRNA	· ROS-triggered TK bonds cleavage induces NP degradation, accelerating mRNA release. · The carrier releases ~70% mRNA and achieves 90% and 85% TIR in CRC and NSCLC, respectively;	24
		Complex NPs	siRNA	· GSH-triggered disulfide bonds cleavage induces NP degradation, accelerating siRNA release; · The carrier releases ~80% siCFL1 within 12 h, silences ~80% CFL1 expression, and achieves >85% TIR in HCC;	25
		Polymeric NPs	RNP	· GSH-triggered disulfide bonds cleavage induces NP degradation, promoting Cas9/sgRNA RNP release; · The carrier releases ~65% Cas9/sgRNA within 12 h in Hepa1-6 cells, downregulates GDF15 protein expression by 69.7%, and achieves >50% gene editing efficiency in HCC;	26
	Enzyme	Polymeric NPs	siRNA	· MMP-triggered sensitive peptide (PLGLAG) cleavage induces NP size shrinkage, enhancing tumor penetration; · The carrier penetrates >90 μm and downregulates PD-L1 mRNA expression by 45% in NCI-H1975 cells;	27
		Biomimetic NPs	miRNA	· MMP-triggered sensitive peptide (PVGLIG) cleavage, accelerating miRNA release; · The carrier achieves ~97% miRNA encapsulation, ~60% release within 12 h, and ~75% TIR in NSCLC;	28
		Hybrid NPs	siRNA	· MMP-triggered sensitive peptide (PVGLIG) cleavage induces CPP exposure, enhancing cellular uptake; · The carrier downregulates PGAM1 mRNA expression by ~60% in A549 cells and achieves 83.5% TIR in NSCLC;	29
		Complex NPs	shRNA	· Esterase-triggered PQDEA hydrolysis induces NP charge conversion, promoting shRNA release; · The carrier downregulates SLC7A11 mRNA expression by ~40% *in vivo* and achieves 77% TIR in HCC;	30
		Complex NPs	siRNA	· Azoreductase-triggered azo bonds cleavage induces NP degradation, promoting siRNA release. · The carrier downregulates PD-L1 mRNA expression by 67% *in vitro* and achieves ~70% TIR in BCa;	31
		Peptide NPs	siRNA	· Furin-triggered sensitive peptide (RVRR) cleavage induces NP degradation, promoting siRNA release. · The carrier downregulates HIF-1α protein expression by 75% *in vitro* and achieves 80% complete tumor elimination in CRC;	32
Exogenous	Light	Complex NPs	siRNA	· NIR-to-UV conversion triggers PPt breakage, accelerating siRNA release; · The carrier releases 73.9% siRNA within 48 h, downregulates 82.7% PLK1 protein expression *in vitro*, and achieves 96.5% TIR in OC;	33
		Hybrid NPs	siRNA	· NIR irradiation increases temperature, inducing liposome phase transition and promoting siRNA release; · The carrier shows 94.89% siRNA encapsulation, 67.87% release efficiency, and 96.6% TIR in PC;	34
		Polymeric NPs	siRNA	· NIR-mediated ROS generation induces endo/lysosomal destruction and TK bonds cleavage, enhancing siRNA cytoplasmic delivery; · The carrier releases ~90% siRNA, enhances ~7-fold endosomal escape, inhibits GPX-4 mRNA expression by 92% *in vitro*, and achieves 91.6% TIR in BCa;	35
	Ultrasound	Lipid NBs	miRNA	· US-mediated nanobubble generation induces NP degradation, promoting miRNA release; · The carrier achieves 88.6% miRNA encapsulation, 70.44% release within 5 h, and 19.32-fold increase in miR-199a-3p expression;	36
		Hybrid NPs	siRNA	· US-mediated BBB opening enhances NPs penetration and uptake in brain tumors; · The carrier exhibits 13-fold increase in brain tumor accumulation, 10-fold increase in siRNA uptake, and 5-fold reduction in SMO protein levels;	37
		Biomimetic NPs	siRNA	· US-mediated ROS production induces endosomal membrane destruction, facilitating endosomal escape; · The carrier enhances endosomal escape by ~2-fold and achieves >80% GPX-4 knockdown efficiency *in vitro*;	38
	Magnetic	Hybrid NPs	siRNA	· Magnetic guidance enhances cellular uptake, resulting in 38% reduction in HER2 mRNA expression *in vitro*;	39
		Hybrid NPs	siRNA	· Magnetic-mediated targeted delivery enhances siRNA accumulation in target tissues; · The carrier achieves ~80% siRNA encapsulation and downregulates VEGF mRNA and protein levels by 60% and 40%, respectively, *in vitro*;	40
		Liposomes	siRNA	· Magnetic-mediated targeted delivery and heat generation enhance siRNA accumulation and release in target tissues; · The carrier achieves 87.12% siRNA encapsulation, ~50% c-Myc mRNA downregulation *in vitro*, and ~60% TIR in BCa;	41
Other stimulus	ATP	LNPs	siRNA	· Elevated ATP binds to PBA, reducing the positive charge of LNPs and promoting siRNA release; · The carrier achieves ~45% MITF mRNA silencing, ~65% apoptosis in B16F10 cells, and ~75% TIR in melanoma;	42
	Hypoxia	Liposomes	siRNA	· Hypoxia-mediated polymetronidazole reduction induces NP degradation, accelerating siRNA release; · The carrier releases 70% siYAP within 14 h, downregulates YAP mRNA expression by 80%, and reduces U87 cell viability by 85%;	43
	miR-21	Complex NPs	RNP	· miR-21 binds to P4, activating the DNAzyme catalytic unit and promoting Cas9/sgRNA RNP release; · The carrier achieves 46.48% miR-21 cleavage, 31.02% apoptosis in HepG2 cells, and 75.94% TIR in HCC;	44
Dual- or multi-responsive	GSH/ATP	Polymeric NPs	siRNA	· Elevated GSH/ATP levels in tumor cells induce NP degradation, promoting siRNA release; · The carrier releases >75% siRNA within 12 h, when combined with BNCT, achieves 95% and 96% TIR in primary and distal BCa, respectively;	45
	pH/GSH	Polymeric NPs	siRNA	· Tumor acidity and high GSH enhance endosomal escape and NP degradation, promoting cytoplasmic siRNA release; · The carrier reduces CD47 mRNA expression by 70% in 4T1 cells and achieves ~95% TIR in BCa;	46
	pH/light/GSH	Polymeric NPs	siRNA	· The system achieves tumor-targeted delivery and spatiotemporally controlled siRNA release in response to pH, light, and GSH;	47
	GSH/HAase/Hypoxia	Complex NPs	RNP	· The system achieves precise and controllable Cas9/sgRNA RNP delivery by sequentially responding to GSH, HAase, and hypoxia in tumors; · The carrier shows 52% mutation frequency of HIF-1α in 4T1 cells and achieves >95% TIR in BCa;	48

ATP: adenosine triphosphate; ATP6: ATP synthase subunit 6; Azo: 4,4'-azodianiline; BCa: breast cancer; BBB: blood-brain barrier; BNCT: boron neutron capture therapy; CA: citraconic anhydride; CDK4/6: cyclin-dependent kinase 4/6; CFL1: cofilin 1; CPP: cell-penetrating peptide; CRC: colorectal cancer; GBM: glioblastoma; GDF15: growth differentiation factor 15; GPX-4: glutathione peroxidase 4; GSH: glutathione; HAase: hyaluronidase; HCC: hepatocellular carcinoma; HER2: human epidermal growth factor receptor 2; HIF-1α: hypoxia-inducible factor-1α; HCQ: hydroxychloroquine; LNPs: lipid nanoparticles; Lipo: lipofectamine; MDK: midkine; MMP: matrix metalloproteinase; MITF: microphthalmia-associated transcription factor; NBs: nanobubbles; NIR: near-infrared; NSCLC: non-small cell lung cancer; OC: ovarian cancer; PC: pancreatic cancer; P4: DNAzyme active region inhibitors; PBA: phenylboronic acid; PBAE: phenylboronic acid pinacol ester; PD-L1: programmed death-ligand 1; PDPA: poly(2-(diisopropylamino)ethyl methacrylate); PEG: polyethylene glycol; PGAM1: phosphoglycerate mutase 1; PLK1; polo-like kinase1; PPt: photoactivatable platinum(IV) (Pt(IV))-backbone polymers; PQDEA: poly N-[2-(acryloyloxy)ethyl]-N-[p-acetyloxyphenyl]-N; RBCm: red blood cell membrane; RNP: ribonucleoprotein; ROS: reactive oxygen species; SAS: squamous carcinoma; shRNA: short hairpin RNA; SLC7A11: solute carrier family 7 member 11; SMO: smoothened; TIR: tumor inhibition rate; TK: thioketal; TME: tumor microenvironment; t_1/2_: half-life; US: ultrasound; UV: ultraviolet; VEGF: vascular endothelium growth factor; YAP: Yes-associated protein.

**Table 3:** Enzyme-responsive strategies for RNA delivery in tumors.

Enzyme	Tumor	Roles in cancer development	Sensitive substrates	Mechanisms of action	Ref.
MMP	Breast cancer Lung cancer Colorectal cancer Prostate cancer Melanoma Glioma Liver cancer Ovarian cancer	ECM degradation; Promote tumor angiogenesis and proliferation; Facilitate tumor invasion and metastasis.	PVGLIG	CPP exposure enhances cellular uptake and tumor penetration.	131
			GPLGVRG	Micelleplex disassembly exposes R9 and enhances cellular uptake.	132
			GPLGIAGQ	Lipid layer degradation exposes positively charged core and promotes cellular uptake.	133
			GPLGLAG	Complex release facilitates deep penetration and cellular uptake.	134
HAase	Breast cancer Prostate cancer Colorectal cancer Bladder cancer Lung cancer	HA degradation, TME modulation; Contribute to tumor progression, invasion, and metastasis.	HA	HA shell degradation exposes cationic PEI core and promotes endo-/lysosomal escape.	135
CTSB	Breast cancer Pancreatic Cancer Liver Cancer Cervical cancer Lung Cancer Colorectal cancer	ECM degradation, protease activation; Promote tumor angiogenesis and proliferation; Facilitate tumor invasion and metastasis; Regulate cell apoptosis and autophagy.	GFLG	Complex disassembly promotes RNA release.	136
			RVRR	Ligand dissociation promotes RNA cytoplasmic delivery.	137
PSA	Prostate Cancer	ECM degradation, immunomodulation; Promote tumor angiogenesis and growth.	HSSKYQ	CPP exposure enhances cellular uptake.	138
Esterase	Liver cancer Melanoma Colorectal cancer Lung cancer Breast cancer Pancreatic Cancer	Regulate cellular metabolism; Enhance tumor cell survival and proliferation; Influence tumor invasion and metastasis.	Ester bond	Charge reversal to negative promotes RNA release.	139
				Carrier disassembly promotes RNA release.	140

CPP: cell-penetrating peptide; CTSB: cathepsin B; ECM: extracellular matrix; HA: hyaluronic acid; MMP: matrix metalloproteinase; PEI: polyethylenimine; PSA: prostate-specific antigen; R9: nona-arginine.

**Table 4:** RNA-based immunotherapy in clinical trials.

Name	Delivery systems	Cargo	Trial	Indication	Phase
mRNA-4157	LNP	INT mRNA	NCT05933577 NCT06077760	Melanoma / NSCLC	III
mRNA-4359	LNP	IDO/PD-L1 mRNA	NCT05533697	Advanced solid tumor	I/II
mRNA-5671	LNP	KRAS neoantigens mRNA	NCT03948763	CRC, NSCLC, pancreatic cancer	I
mRNA-2752	LNP	OX40L/IL-23/IL-36γ mRNA	NCT03739931	Solid tumors / lymphoma	I
mRNA-2416	LNP	OX40L mRNA	NCT03323398	Solid tumors / lymphoma	I/II
MEDI1191	LNP	IL-12 mRNA	NCT03946800	Advanced solid tumor	I
BNT111	LPX	TAA mRNA	NCT04526899	Advanced melanoma	II
BNT112	LPX	TAA mRNA	NCT04382898	Prostate cancer	I/II
BNT113	LPX	HPV-16 mRNA	NCT04534205	Head and Neck Cancer	II
BNT116	LPX	TAA mRNA	NCT05557591	NSCLC	II
BNT122	LPX	Neoantigens mRNA	NCT03815058	Advanced melanoma	II
BNT211	LPX	CLDN6 mRNA	NCT04503278	Solid tumors	I/II
BNT152/153	LPX	IL-7/IL-2 mRNA	NCT04710043	Solid tumors	I
BI1361849	Protamine complex	TAA mRNA	NCT03164772	NSCLC	I/II
STP707	Peptide NPs	TGF-β1/COX-2 siRNA	NCT05037149	Solid Tumors	I
STP705	Peptide NPs	TGF-β1/COX-2 siRNA	NCT04669808 NCT04844983	BCC/isSCC	II

BCC: basal cell carcinoma; CLDN6: claudin-6; COX-2: cyclooxygenase-2; CRC: colorectal cancer; HPV: human papillomavirus; IDO: indoleamine 2,3-dioxygenase; IL: interleukin; INT: individualized neoantigen therapy; isSCC: in situ squamous cell carcinoma; KRAS: kirsten rat sarcoma viral oncogene; LPX: lipoplex; NSCLC: non-small-cell lung cancer; OX40L: OX40 ligand; TAA: tumor-associated antigen; TGF-β1: transforming growth factor-β1.
